# Advanced Nanomaterial-Based Electrochemical Biosensing of Loop-Mediated Isothermal Amplification Products

**DOI:** 10.3390/bios15090584

**Published:** 2025-09-05

**Authors:** Ana Kuprešanin, Marija Pavlović, Ljiljana Šašić Zorić, Milinko Perić, Stefan Jarić, Teodora Knežić, Ljiljana Janjušević, Zorica Novaković, Marko Radović, Mila Djisalov, Nikola Kanas, Jovana Paskaš, Zoran Pavlović

**Affiliations:** 1 BioSense Institute, Research and Development Institute for Information Technologies in Biosystems, University of Novi Sad, Dr Zorana Đinđića 1, 21000 Novi Sad, Serbia; ana.kupresanin@biosense.rs (A.K.); ljsasic@biosense.rs (L.Š.Z.);; 2 Institute of Field and Vegetable Crops, Maksima Gorkog 30, 21000 Novi Sad, Serbia

**Keywords:** GMO, P-35S, P-FMV, oligonucleotide probes, LAMP, 2D nanomaterials, electrochemical biosensors, electrochemical methods

## Abstract

The rapid and sensitive detection of regulatory elements within transgenic constructs of genetically modified organisms (GMOs) is essential for effective monitoring and control of their distribution. In this study, we present several innovative electrochemical biosensing platforms for the detection of regulatory sequences in genetically modified (GM) plants, combining the loop-mediated isothermal amplification (LAMP) method with electrodes functionalized by two-dimensional (2D) nanomaterials. The sensor design exploits the high surface area and excellent conductivity of reduced graphene oxide, Ti_3_C_2_T_x_, and molybdenum disulfide (MoS_2_) to enhance signal transduction. Furthermore, we used a “green synthesis” method for Ti_3_C_2_T_x_ preparation that eliminates the use of hazardous hydrofluoric acid (HF) and hydrochloric acid (HCl), providing a safer and more sustainable approach for nanomaterial production. Within this framework, the performance of various custom-fabricated electrodes, including laser-patterned gold leaf films, physical vapor deposition (PVD)-deposited gold electrodes, and screen-printed gold electrodes, is evaluated and compared with commercial screen-printed gold electrodes. Additionally, gold and carbon electrodes were electrochemically covered by gold nanoparticles (AuNPs), and their properties were compared. Several electrochemical methods were used during the DNA detection, and their importance and differences in excitation signal were highlighted. Electrochemical properties, sensitivity, selectivity, and reproducibility are characterized for each electrode type to assess the influence of fabrication methods and material composition on sensor performance. The developed biosensing systems exhibit high sensitivity, specificity, and rapid response, highlighting their potential as practical tools for on-site GMO screening and regulatory compliance monitoring. This work advances electrochemical nucleic acid detection by integrating environmentally-friendly nanomaterial synthesis with robust biosensing technology.

## 1. Introduction

Genetically modified organisms (GMOs) are organisms whose genetic material has been intentionally altered using modern genetic engineering techniques to insert, delete, or modify specific genes in ways not achievable through natural reproduction or traditional breeding. Commonly employed methods include recombinant DNA technology, which allows for the insertion of genes from one organism into another to confer desirable traits such as pest or herbicide resistance [1]; gene editing technologies such as CRISPR-Cas9, which enable precise DNA modifications [2]; and RNA interference, which inhibits the expression of specific genes to enhance viral resistance [3]. These technologies have been applied to produce a range of GM crops, such as *Bt* cotton and golden rice, offering benefits including increased yield, enhanced resistance to biotic and abiotic stresses, improved nutritional profiles, and reduced dependence on chemical inputs [4,5]. Despite their advantages in addressing food security, climate resilience, and malnutrition, GMOs remain the subject of public and scientific debate due to concerns over potential health risks, environmental impacts, and ethical considerations [6].

The global use and regulatory debates surrounding GMOs necessitate accurate, high-throughput detection methods for compliance, labeling, and traceability. To achieve this, a variety of analytical strategies have been developed, broadly categorized into DNA-based and protein-based approaches [7,8,9]. DNA-based methods, particularly polymerase chain reaction (PCR), are the most extensively used due to their high sensitivity, specificity, and adaptability. Variants such as real-time PCR allow both qualitative and quantitative analyses, while multiplex PCR enables the simultaneous detection of multiple genetic elements [10]. Emerging molecular techniques such as digital PCR, loop-mediated isothermal amplification (LAMP), and next-generation sequencing (NGS) further enhance accuracy, scalability, and resolution [9].

At the core of all GMO detection strategies is the identification of genetic elements introduced during the modification process. The most commonly targeted sequences include promoters (e.g., cauliflower mosaic virus 35S promoter—P-35S, figwort mosaic virus promoter—P-FMV), terminators (e.g., *Agrobacterium tumefaciens* nopaline synthase terminator—T-nos), selectable marker genes (e.g., genes encoding aminoglycoside 3′-adenyltransferase–*aadA*), neomycin phosphotransferase II—*nptII*, and β-glucuronidase—*uidA*), and trait genes conferring herbicide or insect resistance (e.g., 5-enolpyruvylshikimate-3-phosphate synthase gene—*EPSPS*, insecticidal crystal protein-coding genes—*cry* genes). These elements are selected due to their frequent incorporation into transgenic constructs and their distinct sequence signatures, which facilitate their detection across various crop species and product types [11]. The detection methods vary in specificity, ranging from screening approaches that target common regulatory elements to the most precise event-specific methods that identify unique transgene insertion sites in the host genome [12].

In recent years, LAMP has emerged as a promising alternative to conventional PCR, offering rapid, highly specific, and sensitive detection of common transgenic elements such as P-35S, P-FMV, T-nos, and several marker genes, as well as event-specific detection [11,13,14,15,16]. The LAMP method operates at a constant temperature (60–65 °C), eliminating the need for thermal cycling. The method relies on the strand-displacing activity of the large fragment of *Bst* DNA polymerase, derived from *Geobacillus stearothermophilus*, and a set of four to six primers that target six to eight distinct regions of the DNA sequence. The reaction begins with the annealing of inner (FIP/BIP) and outer (F3/B3) primers, which initiates the formation of a dumbbell or stem-loop structure that serves as a template for subsequent amplification. The addition of loop primers (LoopF/LoopB) further accelerates the reaction by binding to the looped regions, enhancing amplification speed and product yield. As a result, the LAMP reaction can generate up to 10^9^ copies of DNA within an hour, often forming large concatemeric products with “cauliflower-like” structures [17,18,19]. Reaction monitoring can be achieved via multiple readout strategies, including turbidimetry, fluorometry, and colorimetry, making LAMP compatible with both laboratory and field diagnostics [19]. Furthermore, the method’s tolerance to PCR inhibitors and the use of simple heating devices such as heat blocks make it particularly well-suited for low-resource and point-of-need (PoN) settings [20].

Nanomaterials boost sensitivity, selectivity, and miniaturization of electrochemical DNA biosensors through superior electron transfer and signal amplification [21,22]. Gold nanoparticles (AuNPs) are widely used for their conductivity, biocompatibility, and probe immobilization capacity [23,24,25]. Metal oxides like ZnO and TiO_2_ enhance stability and conductivity [26]. Carbon nanotubes (CNTs) improve charge transport and lower detection limits [23,24,25,27], while graphene/GO provides high-density DNA probe anchoring [24,27]. Emerging 2D materials, including MXenes (e.g., Ti_3_C_2_T_x_) and TMDCs, further enhance performance via tunable surface chemistry and high conductivity [28,29,30,31,32].

Effective DNA probe immobilization on nanomaterials is crucial for developing sensitive electrochemical biosensors. Thiol-modified probes form stable Au-S bonds on gold surfaces, while amine-terminated probes covalently attach to carboxyl-functionalized substrates [32,33,34,35]. Carbon nanomaterials like CNTs and graphene enable immobilization through π–π stacking, electrostatic interactions, or covalent EDC/NHS coupling, offering flexibility between regenerability and stability [36,37]. Optimal probe density is essential—excessive crowding causes steric hindrance, while insufficient coverage reduces target accessibility [30,38,39]. Surface passivation with blocking agents (e.g., BSA, mercaptohexanol) minimizes non-specific binding [38,39].

Recent advances employ both signal-on and signal-off detection mechanisms. Signal-off sensors measure reduced Faradaic current upon hybridization [40,41], enhanced via enzymatic recycling or DNA nanostructures [32,42,43,44,45]. While simple and regenerable, they require amplification for ultrasensitive detection. Signal-on systems generate current increases through target-induced redox tag movement, offering higher signal gains (100–700%) but sometimes limited reusability [42,46,47,48,49,50].

Probe density critically impacts performance, with 10^12^–10^13^ molecules/cm^2^ (200 nM–1 µM immobilization concentration) being typically optimal. Excessive density causes steric hindrance, while insufficient coverage yields weak signals [51,52,53,54,55,56,57,58]. Statistical optimizations suggest that 0.5 µg/mL (~75–150 nM) often balances hybridization efficiency and signal response [57].

The objectives of this study was (1) to validate LAMP primers for the detection of regulatory elements of transgenic constructs in plants (P-35S, P-FMV, and T-nos); (2) to explore electrochemical-based detection of LAMP products using different kinds of electrodes functionalized with 2D nanomaterials (reduced graphene oxide, MXenes, and MoS_2_) and AuNPs; and (3) to evaluate and compare the efficiency of different types of oligonucleotide DNA probes to hybridize with target DNA (LAMP products) using diverse biosensing systems, i.e., combination of electrodes, 2D nanomaterials, and electrochemical detection methods.

## 2. Materials and Methods

### 2.1. Chemicals and Commercial Materials Used

WarmStart LAMP Kit (DNA and RNA) (New England Biolabs, MA, USA), Chelex-100 resin (Bio-Rad, CA, USA), 60% of polyethylene glycol 200 (PEG 200) (Sigma-Aldrich Co. LLC, Darmstadt, Germany), 20 mM NaOH (Sigma-Aldrich, St. Louis, MO, USA), gBlocks^®^ DNA Fragments (Integrated DNA Technologies, IA, USA), graphene oxide, 2 mg/mL dispersion in H_2_O (Sigma-Aldrich, St. Louis, MO, USA), DI water, 1-Ethyl-3-(3-dimethylaminopropyl)carbodiimid-hydrochlorid (EDC-HCl) (Carl Roth, Karlsruhe, Germany), N-Hydroxysuccinimide Solid, 98% Poly bottle (Sigma-Aldrich, St. Louis, MO, USA), Cysteamine ≥ 98.0% (RT) (Sigma-Aldrich, St. Louis, MO, USA), 1-methyl-2-pyrrolidinone (Sigma-Aldrich, St. Louis, MO, USA), Phosphate Buffered Saline 10X Solution (Fisher Scientific, Portsmouth, NH, USA), Gold(III) chloride hydrate 99.995% trace metals basis (Sigma-Aldrich, St. Louis, MO, USA), Sulfuric acid (H_2_SO_4_) 99.999% (Sigma-Aldrich, MO, USA), Tris(2-carboxyethyl)phosphine hydrochloride powder ≥ 98% (Sigma-Aldrich, MO, USA), 6-Mercapto-1-hexanol 97% (Sigma-Aldrich, MO, USA), Molybdenum(iv) sulfide, nanopowder 90 nm diameter (APS) 99% trace metals basis (Sigma-Aldrich, St. Louis, MO, USA), Bovine Serum Albumin Fraction V protease-free (Sigma-Aldrich, St. Louis, MO, USA), Tween™ 20 Fisher BioReagents (Fisher Scientific, Portsmouth, NH, USA), (3-Aminopropyl)triethoxysilane, 99% (Sigma-Aldrich, St. Louis, MO, USA), Hydrogen peroxide solution, 30% in H_2_O, ACS reagent (Sigma-Aldrich, St. Louis, MO, USA), Acetic acid 99% (Sigma-Aldrich, St. Louis, MO, USA), Mes sodium salt ≥ 99% (Sigma-Aldrich, St. Louis, MO, USA), Titanium aluminum carbide MAX phase (Ti_3_AlC_2_) (Nanochemazone, Alberta, Canada), Citric acid anhydrous crystalline (Fisher Scientific, Portsmouth, NH, USA), dimethyl sulfoxide DMSO BioReagent ≥ 99.9% Molecular Biology Grade liquid (Fisher Scientific, Portsmouth, NH, USA), ammonium bifluoride NH_4_HF_2_ (Fisher Scientific, Portsmouth, NH, USA).

### 2.2. DNA Preparation

#### 2.2.1. gBlocks Design

All molecular analyses were done using gBlocks^®^ DNA Fragments (Integrated DNA Technologies, Newark, NJ, USA), synthetic dsDNA strings representing fragments of target elements often present as part of transgenic constructs in GM plants. They correspond to fragments of P-35S, P-FMV, and T-nos and were designed using sequences of the cauliflower mosaic virus genome (GenBank acc. no. V00141), figwort mosaic virus genome (GenBank acc. no. NC_003554), and *Agrobacterium tumefaciens* gene encoding nopaline synthetase (GenBank acc. no. V00087), respectively (Table S1).

#### 2.2.2. Plant DNA Isolation

Plant DNA was isolated from soybean (*Glycine max*), rapeseed (*Brassica napus*), and wheat (*Triticum aestivum*) seed and leaf tissues using a crude extraction protocol that combines the Chelex method with an alkaline PEG lysis buffer, whose composition follows the formulation described by Tomlinson and Boonham [59]. For each sample, 125 mg of plant tissue was placed into a 5 mL microcentrifuge tube containing 0.4 g of Chelex-100 resin (Bio-Rad, Hercules, CA, USA), one sterilized metal bead (10 mm in diameter), and 900 µL of alkaline PEG lysis buffer pH 13.3–13.5: 60% of polyethylene glycol 200 (PEG 200) (Sigma-Aldrich Co. LLC, Darmstadt, Germany), 20 mM NaOH (Sigma-Aldrich, St. Louis, MO, USA). The tube was shaken vigorously for 1 min to ensure mechanical disruption of the tissue, followed by incubation at room temperature for 20 min to facilitate DNA release and allow Chelex resin to settle and remove impurities. After incubation, a 20 µL aliquot of the supernatant was then diluted 1:50 with 1 mL of dilution buffer (1× TE buffer, pH 8). The diluted extract was used to prepare “mock” GM DNA for subsequent LAMP assays.

#### 2.2.3. “Mock” GM DNA Samples

The resulting crude DNA extracts were quantified using the BioSpec-nano spectrophotometer (Shimadzu, Kyoto, Japan) and diluted to a working concentration of 1 ng/μL in molecular-grade water. Each plant DNA sample was then spiked with synthetic gBlock DNA (either P-35S or P-FMV; 0.001 ng/μL) to simulate GM material. Final samples containing 1 µL of gBlock DNA and 9 µL of plant DNA constituted a final GM DNA proportion of approximately 0.0111% (111 ppm) relative to total DNA content.

#### 2.2.4. LAMP Assay

LAMP was performed using six-primer sets targeting regulatory sequences of the transgenic construct, i.e., P-35S [60], P-FMV [13], and T-nos [61] (Table S1). The 25 µL reaction mixture contained the WarmStart LAMP 2× Master Mix from New England Biolabs (Ipswich, MA, USA), 5× primer mix (8 µM each of FIP/BIP, 1 µM each of F3/B3 primers, 2 µM each of LoopF/LoopR primers), LAMP fluorescent dye (50×) (New England Biolabs, Ipswich, MA, USA), molecular-grade water, and 1 µL target DNA. The reactions were prepared following the WarmStart LAMP Kit (DNA and RNA) (New England Biolabs, Ipswich, MA, USA) protocol using 5× instead of 10× LAMP primer mix. LAMP reactions were carried out in triplicate, using the Genie III Instrument (OptiGene, Horsham, UK) at 65 °C for 30 min.

The limit of detection (LOD) for each region was determined by testing 10-fold serial dilutions of the purified template (gBlocks) prepared in molecular-grade water, ranging from 0.1 ng/μL to 1 × 10^−6^ ng/µL. Stock concentration of gBlocks used for dilution preparation was 10 ng/μL. The LOD was determined empirically as the lowest concentration at which all technical replicates produced a positive amplification signal.

To simulate LAMP testing of real samples, “mock” GM DNA samples were used.

For electrochemical analysis, LAMP reactions were prepared with incubation times of 30 min at 65 °C using a 5× primer mix and without the LAMP fluorescent dye (50×) (New England Biolabs, Ipswich, MA, USA). We used 10^–3^ ng/μL and 10^–4^ ng/μL P-35S and P-FMV gBlocks as reaction templates, respectively, for electrochemical detection optimization, while for the electrochemical calibration curve, 10-fold serial dilutions ranging from 1 × 10^−3^ ng/µL to 1 × 10^−7^ ng/µL were used as reaction templates. Rapeseed leaf DNA spiked with a P-35S gBlock, simulating a real GM sample, was used for the validation of the electrochemical detection. Reactions were prepared fresh on the day of electrochemical measurement to ensure consistency and minimize the degradation of amplification products.

#### 2.2.5. Oligonucleotide Probe Design

Oligonucleotide DNA probes (Table 1, Table S1) were designed using the online tool OligoAnalyzerTool (available at https://www.idtdna.com/pages/tools/oligoanalyzer, accessed on 24 March 2025). Oligonucleotide DNA probes were designed to be in the range of 20–45 nucleotides with G–C content of 40–60%, no more than four bases in a row, and with G or C at the end [62]. Additionally, the position of the DNA probe in relation to the target LAMP amplicon sequence was in the second and/or third quarter from the 5’ end of the target sequence (Figures S1 and S2). We have tested probe sequences for self-dimer and hairpin formation, as well as for heterodimer formation with LAMP primers. Suitably, we have chosen sequences that show ΔG > −9.0 kcal/mol as recommended for designing oligonucleotide primers and probes [63]. In the case of heterodimers, we also checked that complementarity with primer sequences is less than five nucleotides in a row.

**Figure 1:**
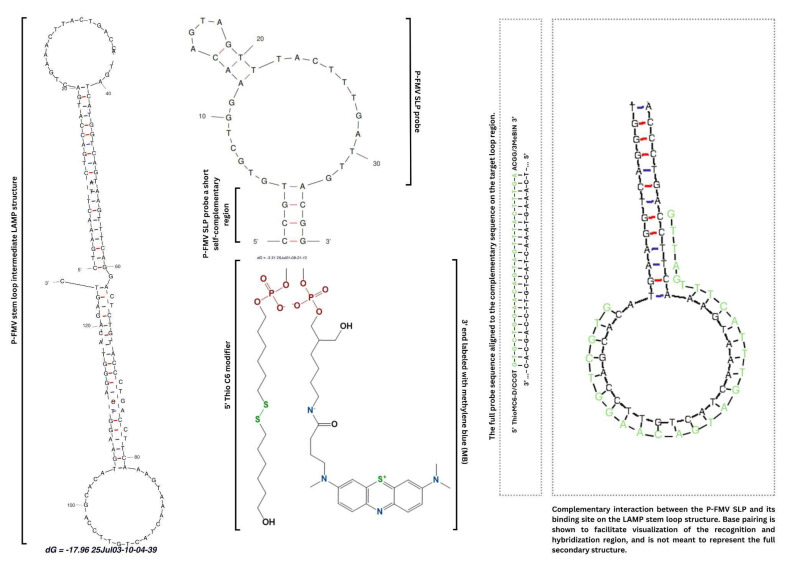
Hybridization mechanism of the P-FMV stem-loop DNA probe to the complementary sequence fragment of the P-FMV amplification product of LAMP reaction. The DNA secondary structure of the LAMP product was predicted using the Mfold Web Server for nucleic acid folding and hybridization prediction [66]. Chemical structures were drawn using Chemdraw v22.0, and sequence alignment analysis was performed with the VectorBuilder Sequence Alignment Tool [67].

Modification of oligonucleotide DNA probes was done by consulting relevant literature [42,64,65] and is listed in Table 1. Schematic representation of the hybridization between probe and template DNA—amplification product of LAMP reaction, is shown in Figure 1.

### 2.3. Nanomaterials Synthesis and Preparation

#### 2.3.1. Green Synthesis of Ti_3_C_2_T_x_

**Figure 2:**
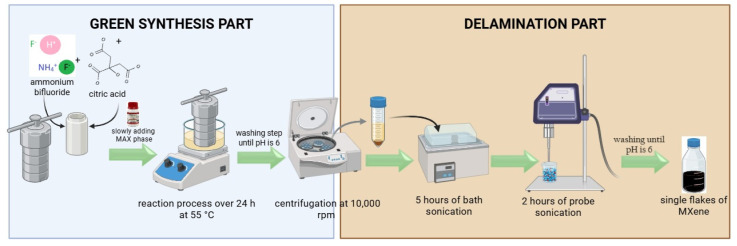
Synthesis and delamination pathway of obtaining Ti_3_C_2_T_x_ MXene via green synthesis method.

The Ti_3_C_2_T_x_ MXene was synthesized via a green etching strategy [68] designed to promote the formation of surface –COOH functional groups, which are essential for subsequent bioconjugation with amino-functionalized biomolecules. The aluminum atomic layer was selectively removed from the Ti_3_AlC_2_ MAX phase using a mild etching mixture composed of citric acid (6 M) and ammonium bifluoride (6 M). The etching process was carried out in a standard Teflon-lined autoclave at 55 °C for 24 h. Following the reaction, the obtained material was thoroughly washed with deionized water until a neutral pH was reached, followed by intercalation steps to achieve adequate delamination and increase the interlayer spacing of the flakes (Figure 2). As there were no earlier reports on MXene synthesis using this specific etching agent combination, the approach was inspired by the concept of in situ HF generation under hydrothermal conditions. Citric acid and ammonium hydrogen fluoride are both water-soluble and were therefore dissolved in deionized water to form in situ HF necessary for etching of MAX phase. The concentration of 6 M was chosen as the set point for both reagents, aiming to strike a balance between etching efficiency and material stability, i.e., sufficiently reactive to remove the Al layer from the MAX phase but not too aggressive to cause degradation of the resulting Ti_3_C_2_T_x_ MXene. The synthesis results confirmed that this concentration was optimal for successful MXene formation while preserving the fundamental structure of the material. This synthetic route offers a safer and more environmentally friendly alternative to conventional HF-based methods, while simultaneously introducing carboxyl (-COOH) terminal groups favorable for subsequent covalent functionalization [69].

The synthesized Ti_3_C_2_T_x_ powder (1 g total) was dispersed in DMSO for delamination. A sequential sonication protocol (5 h bath + 2 h probe sonication) achieved effective interlayer separation, yielding few-to single-layer nanosheets (Figure 2). The dispersions were then washed with deionized water to pH ≈ 6 and centrifuged to remove unexfoliated particles. The supernatant, containing predominantly single-layer flakes, underwent additional centrifugation for purification. Both final supernatants and sediments were stored under argon at 4 °C to prevent oxidation and maintain colloidal stability.

#### 2.3.2. MoS_2_ Exfoliation

We prepared few-layer MoS_2_ nanosheets through ultrasonic exfoliation of polycrystalline MoS_2_ powder (100 mg) in isopropanol (10 mL) using a Bandelin 70HD homogenizer (BANDELIN electronic GmbH & Co. KG, Berlin, Germany) operating at 70 W (30% power output). Isopropanol was chosen as the solvent due to its optimal surface energy matching with MoS_2_. The exfoliation process, conducted in an ice bath to maintain temperature, was performed for three different durations (10 min, 1 h, and 3 h) to evaluate time-dependent effects. Following initial sonication, the dispersion underwent centrifugation at 5000 rpm for 5 min to remove unexfoliated aggregates. The sediment was then resuspended in fresh isopropanol and subjected to a second sonication step (10 min) followed by final centrifugation at 7500 rpm for 10 min to isolate the desired few-layer nanosheets. This optimized protocol enables precise control over flake size and layer thickness through systematic variation of sonication and centrifugation parameters.

#### 2.3.3. Materials Characterization

Morphology of the obtained 2D nanomaterials was investigated using Apreo 2C Scanning Electron Microscope (Thermo Fisher Scientific, Waltham, MA, USA). The structural properties were analyzed using the Rigaku SmartLab X-ray diffractometer (Rigaku Corporation, Akishima-shi, Tokyo, Japan), equipped with a high-flux 9 kW PhotonMax rotating anode X-ray source (Cu/Mo) and a HyPix-3000 semiconductor detector for high-resolution measurements in 0D, 1D, and 2D modes. XPS analysis was performed using a SPECS instrument (custom-made) for surface characterization. Photoelectron emission was excited by the monochromatic Al Kα line with photon energy of 1486.67 eV. Micro-Raman measurements were performed with the Horiba Explora Plus system (Palaiseau, France) equipped with a 532 nm laser excitation source, 1200 g/mm grating, and 100× magnification objective. Porosity measurements were performed on the NOVAtouch XL2 Series (Anton Paar GmbH, Graz, Austria) using N_2_ gas as the probe molecule, with Brunauer-Emmett-Teller (BET) analysis for surface area determination.

### 2.4. Electrode Preparation, Various Nanomaterial Surface Functionalization, and Electrochemical Signal-Measurement Methodology

#### 2.4.1. Fabrication of In-House Electrodes

Gold-leaf electrodes were produced according to the procedure described elsewhere [70]. Briefly, 24-karat gold leaf sheets (80 mm × 80 mm) are laminated onto a four-layer PVC adhesive substrate (4 × 125 µm) that had been pre-coated with PTFE spray to render the surface hydrophobic. The gold leaf was adhered to the PVC using light pressure at 180 °C in a hot laminator. Electrode patterns, including a 3 mm diameter circular working electrode, were created by laser ablation with an Nd:YAG Power Line D-100 laser (hatch mode, 26.2 A, 65 kHz, 500 mm/s).

PVD gold electrodes are fabricated using a PVD system Leybold Heraeus L560Q (Dresden, Germany), via an e-beam evaporation of chromium buffer layer (100 nm thickness) and gold (100 nm thickness) on a PVC substrate using a custom-made shadow mask for direct electrode patterning. Chromium is deposited with the following parameters: theoretical density 7.2 g/cm^3^, Z-ratio 0.305, geometrical factor 1, power of e-beam 40, avg. deposition speed 2.1 Å/s; for gold deposition, the parameters are as follows: theoretical density 19.8 g/cm^3^, Z-ratio 0.381, geometrical factor 1, e-beam power 120, avg. deposition speed 1.6 Å/s.

#### 2.4.2. Modification of Electrode Surface

All gold working electrodes were 3.0 mm in diameter and were first electrochemically cleaned in 0.5 M H_2_SO_4_ by cyclic voltammetry (potential range −0.5 to 1.50 V vs. saturated Ag/AgCl; scan rate 300 mV/s; 10 cycles) before further functionalization and immobilization.

Gold nanoparticles were electrodeposited onto several types of gold-working electrodes by chronoamperometry. Each electrode was immersed in an aqueous electrolyte containing 0.5 mM HAuCl_4_ and 0.5 M H_2_SO_4_, and a constant potential of −0.5 V versus a saturated Ag/AgCl reference was applied for 10 min. Using the same electrolyte, AuNPs were likewise deposited onto carbon electrodes by applying −1.0 V versus saturated Ag/AgCl for 10 min. A linear thiol-terminated oligonucleotide DNA probe was immobilized onto gold-nanoparticle-decorated electrodes of five types: PVD-coated gold, commercial screen-printed gold (Zensor AUTE100), laser-patterned gold leaf, screen-printed sintered gold (800 °C), and an AuNP-modified carbon electrode (Zensor TE100) via spontaneous Au–S chemisorption during a 2 h incubation. For blocking step, 1 mM 6-mercapto-1-hexanol prepared in 1 × PBS was drop-casted onto the working electrode and kept for 1 h. The electrodes were subsequently thoroughly rinsed with deionized water for 30 s and stored in 1 × PBS in the fridge at 4 °C until use.

The Ti_3_C_2_T_x_ MXene was prepared through citric acid treatment followed by repeated centrifugation and washing with deionized water until reaching neutral pH. Mechanical delamination was achieved by freezing the suspension, exploiting water expansion to increase interlayer spacing, followed by thawing and purification. For electrode deposition, we employed a modified Langmuir–Blodgett technique where a gold electrode was vertically immersed in deionized water. After applying a Ti_3_C_2_T_x_ suspension to the water surface, controlled drainage through a bottom valve enabled uniform deposition as the electrode surface emerged.

Prior to graphene oxide deposition, gold electrodes were modified with a cysteamine self-assembled monolayer (10 mM in deionized water, 1 h incubation). Graphene oxide (GO) suspensions (0.2 mg/mL in 50:50 water/N-methyl-2-pyrrolidone) were activated with EDC/NHS (2 mM/5 mM, 2 h) before drop-casting onto the functionalized PVD gold and commercial AUTE100 electrodes. After 2 h adsorption and N_2_ drying, the electrodes underwent thermal annealing (100 °C, 30 min) followed by electrochemical reduction of GO via cyclic voltammetry (20 cycles, −0.4 to −1.2 V at 25 mV/s in PBS) to produce stable reduced GO (rGO)-modified electrodes [71].

The amine-terminated linear DNA probe was covalently immobilized on three distinct electrode platforms: (i) a gold electrode coated with rGO; (ii) a gold electrode modified with green-synthesized, carboxyl-rich MXene nanosheets; and (iii) a commercial graphene electrode bearing surface -COOH groups. In each case, the probe’s terminal -NH_2_ groups reacted with carboxyl groups on the electrode surface, forming a stable amide linkage. The carboxyl groups of both MXene and graphene/rGO were activated by drop-casting 0.2 M EDC/0.05 M NHS prepared in 0.1 M MES buffer (pH 5.0) and incubated for 2 h. After thorough rinsing with deionized water, a 5 µL aliquot of amine-terminated DNA probe (surface density of 750 pmol/cm^2^) was applied to the electrode and allowed to react for 2 h to form amide bonds. The electrode was again rinsed with water and dried gently.

For the MXene-modified electrode, a blocking step with 100 mM ethanolamine in deionized water (1 h) was performed to minimize non-specific adsorption. For the electrodes modified with reduced GO, two alternative blocking strategies were evaluated: (i) incubation with bovine serum albumin (3% BSA with 0.1% Tween 20 in 1 × PBS, pH 7.2, 1 h) and (ii) quenching with 100 mM ethanolamine in deionized water (1 h). The blocking step for the commercial carboxylated graphene electrode comprised a 1 h incubation in 100 mM ethanolamine prepared in deionized water.

To functionalize the gold electrode with molybdenum disulfide, the gold surface was first oxidized to form a thin Au-oxide layer. Oxidation was achieved by cyclic voltammetry in 0.1 M acetic acid, where the potential was swept for 20 cycles at 100 mV/s. The scan was stopped at the end of the twentieth cycle, after the appearance of the gold-oxide peak. A 2% solution of (3-aminopropyl)triethoxysilane (APTES) was prepared in absolute ethanol and drop-casted onto the oxidized gold working electrode for 1 h. The electrode was then rinsed with ethanol and baked in an oven at 110 °C for another 1 h. MoS_2_ powder was treated with 3% hydrogen peroxide for 10 min, washed thoroughly with deionized water, and redispersed to a concentration of 5 mg/mL. This suspension was drop-casted onto the APTES-modified electrode and allowed to incubate for 12 h before use. A linear oligonucleotide DNA probe bearing a terminal thiol (-SH) group was immobilized on the MoS_2_-modified electrode via spontaneous thiol bond formation during a 2 h incubation. After thorough rinsing, residual active sites were passivated by treating the electrode with 1 mM 6-mercapto-1-hexanol (MCH) in 1 × PBS for 1 h.

The oligonucleotide probe concentration used during the immobilization step was, in most experiments, 525 μmol/dm^3^ (surface density 750 pmol/cm^2^). Electrochemical experiments were also conducted using a higher and lower probe concentrations (1050 μmol/dm^3^, surface density 1500 pmol/cm^2^, and 105 μmol/dm^3^, surface density 150 pmol/cm^2^).

**Figure 3:**
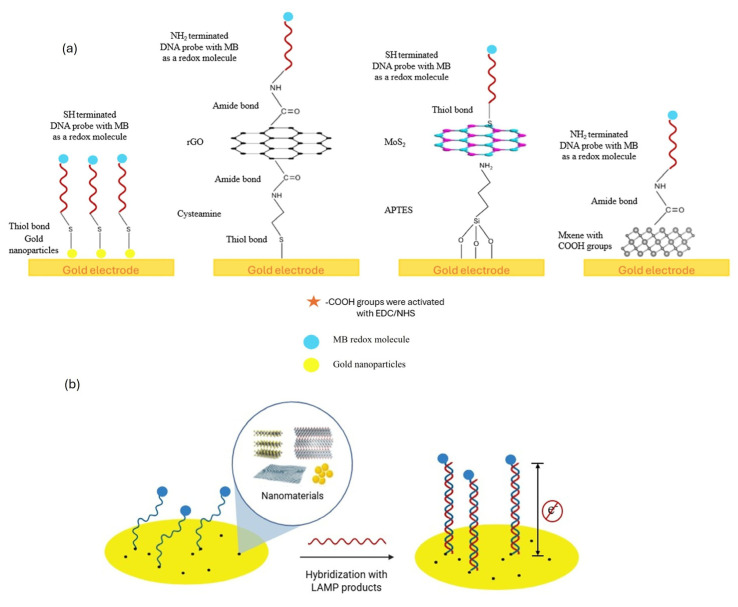
(**a**) Schematic representation of gold electrode functionalization with advanced nanomaterials (AuNPs, rGO, MoS_2_, MXene) enabling DNA probe immobilization, (**b**) Detection mechanism after hybridization of the target LAMP products with immobilized DNA probes and much slower electron transfer kinetics of the MB redox molecules.

Nanomaterials can enhance electrochemical DNA detection in several ways. Their surface roughness increases the available electrochemically active area, allowing more DNA probe molecules to attach to the working electrode and facilitating greater electron transfer. The shape and density of nanoparticles can also affect the efficiency of DNA probe binding. Additionally, nanoparticles may facilitate the favorable reorganization of DNA molecules, thereby enhancing target recognition. The electronic conductivity of different nanomaterials can influence the rate of electron transfer, while their varying electrocatalytic properties can affect the signal intensity of redox probes like methylene blue (MB). As illustrated in Figure 3a, the gold electrode platform was engineered through the integration of diverse nanomaterials (AuNPs, rGO, MoS_2_, MXene), creating multifunctional interfaces that facilitated the immobilization of DNA probes and improved the analytical performance of the biosensing system. Figure 3b shows the construction of the working electrode using various nanomaterials and DNA detection mechanism.

#### 2.4.3. Electrochemical Signal Measurement

Five electrochemical techniques for DNA target detection were subsequently evaluated: square-wave voltammetry (SWV), differential-pulse voltammetry (DPV), linear-sweep voltammetry (LSV), alternating-current voltammetry (ACV), and electrochemical impedance spectroscopy (EIS). Each electrode was first recorded in 1 × PBS (some electrodes were also tested in 0.1 × PBS) to obtain a baseline, then re-recorded after target addition. The target DNA (amplification product of LAMP reaction) was diluted in 1 × PBS, thermally denatured at 95 °C for 5 min, and snap-cooled on ice for 2 min to prevent re-annealing. Hybridization with the surface-bound oligonucleotide DNA probe proceeded for 1 h. Among the techniques tested, SWV gave the best analytical performance; optimal SWV parameters were pulse amplitude 75 mV, pulse width 2 ms, step potential 10 mV, and potential window −0.6 to −0.1 V vs. saturated Ag/AgCl. EIS was employed only for linear oligonucleotide DNA probes lacking MB. Baselines were recorded in 10 mM Fe(CN)_6_^3−^/^4−^ (1:1) containing 0.1 M KCl (frequency range 1 Hz–100 kHz; AC amplitude 10 mV). After hybridizing the denatured target (1 h, as above) and rinsing the electrode with deionized water, a second EIS measurement was acquired in the same redox electrolyte to evaluate charge-transfer resistance changes upon target binding.

All of the electrochemical experiments were conducted by Biologic SP-300 potentiostat, (Seyssinet-Pariset, France).

## 3. Results and Discussion

One of the initial steps in the conducted research was the evaluation of the LAMP reaction, including primer validation, concentration of primers (5× and 10× primer mix, see Section 2.2.4), and amplification time, in order to ensure robust and specific detection of regulatory elements of transgenic constructs (P-35S, P-FMV, T-nos). Additionally, oligonucleotide probe design was performed as a separate step to further enhance detection specificity. Parallel to this, advanced 2D nanomaterials, such as rGO, Ti_3_C_2_T_x_ and MoS_2_, were synthesized and functionalized to enhance biosensor performance. These nanomaterials were tailored for high DNA binding affinity and efficient signal transduction, enabling sensitive and selective detection. The developed platform successfully coupled LAMP-amplified DNA targets with functionalized nanomaterial biosensors, enabling rapid GMO detection.

### 3.1. Determination of LAMP LOD

The limit of detection testing revealed sensitivities of approximately 4 fg/µL (≈0.1 pg per reaction) for P-FMV, 40 fg/µL (1.0 pg per reaction) for P-35S, and 0.4 pg/µL (10 pg per reaction) for T-nos, aligning with previously reported LODs in LAMP assays for GMO detection (Figure S3).

During primer evaluation and LOD determination, the T-nos target region showed suboptimal LAMP performance under the tested conditions. Amplification was consistently delayed compared with other target regions, often exceeding the ideal 20 min LAMP reaction time, and results were inconsistent across replicates. While such delays may be acceptable in a conventional PCR, they are suboptimal for LAMP, where speed and consistency are essential. It is presumed that the suboptimal performance was related to the temperature parameters recommended by the commercial kit. Based on literature reports [61], further optimization of the reaction, specifically for the detection of the T-nos regulatory element, may be achieved by lowering the reaction temperature. Due to poor reproducibility, the T-nos region was excluded from further testing on gBlock-spiked plant DNA samples and subsequent electrochemical detection experiments.

### 3.2. Analytical Sensitivity of LAMP in the Context of GMO Regulations

According to EU legislation 1829/2003 and 1830/2003, labeling is not required for conventional or organic food and feed products that contain authorized GMOs at levels below 0.9%, provided such presence is adventitious or technically unavoidable [72]. In contrast, the presence of unauthorized GMOs is strictly prohibited in the EU, regardless of the concentration, including levels below the 0.9% threshold [73]. Nevertheless, products containing GMO material below this threshold are still considered GMO-containing food, which has raised concerns regarding transparency and trace-level exposure. EU member state laboratories routinely conduct sampling and qPCR-based assays to monitor compliance, and in Germany, national testing over 2017–2021 detected labeling-level GMO content (>0.9%) in only 0.1–0.2% of soybean and maize samples, demonstrating the regulatory system’s effectiveness [74]. On the other hand, regulatory enforcement and threshold definitions vary globally. In many non-EU countries, GMO labeling policies are less stringent or inconsistently applied, resulting in limited consumer protection and challenges in cross-border regulatory harmonization.

Given the stringent regulatory landscape in the EU and the variability of GMO labeling policies worldwide, there is a critical need for sensitive and rapid detection methods to ensure compliance and consumer protection. In this context, the LAMP assay presented in our study demonstrated exceptional analytical sensitivity, capable of detecting GMO content well below the 0.9% labeling threshold (Figure S4), thereby supporting effective monitoring and enforcement efforts.

We demonstrated the capability of the LAMP assay to achieve femtogram detection levels corresponding to 0.1% and even 0.01% GMO content (GM DNA proportion of approximately 0.0111% or 111 ppm relative to total DNA content; Figure S4), substantially surpassing the 0.9% GMO content threshold required for mandatory labeling under EU regulations, highlighting the analytical sensitivity of the assay for both regulatory compliance and trace-level screening applications. These results are in line with previously reported limits of detection (LODs) in LAMP assays for GMO detection. For example, Singh et al. [75] developed a multiplex real-time LAMP assay capable of simultaneously detecting the P-FMV promoter, the *nptII* marker gene, and the P-35S*-cry1Ac* construct region, with detection limits as low as 0.1% for each target within 45 min. Takabatake et al. [76] developed LAMP-based methods for GM maize and soybean with detection limits of ≤ 0.5% using direct sample preparation, while Xing et al. reported integrated PAMMPs@DNA-LAMP assays achieving 0.01% sensitivity within 40 min [14]. Further improvements have coupled LAMP with CRISPR/Cas12a [77] or TaqMan probes [78] to reach detection limits as low as five copies in under 20 min. To simulate a realistic GM environment, we spiked synthetic double-stranded DNA fragments (gBlocks) representing transgenic elements into genomic DNA isolated from soybean (*Glycine max*), rapeseed (*Brassica napus*), and wheat (*Triticum aestivum*) seed and leaf tissues. This approach provided a controlled and reproducible method for evaluating assay sensitivity across different crop types and tissue sources. The successful detection across all tested plant matrices confirms the robustness and versatility of both the Chelex-based DNA extraction method and the LAMP assay system, which demonstrated excellent analytical performance in terms of sensitivity, speed, and tolerance to crude DNA extracts. Collectively, these findings show that our LAMP assay is consistent with state-of-the-art GMO detection strategies, demonstrating its practical suitability for rapid, sensitive, and on-site applications.

### 3.3. Nanomaterials Characterization

#### 3.3.1. MXene Characterization

**Figure 4:**
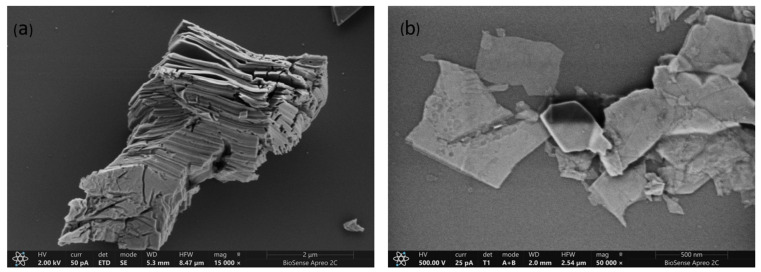
HRSEM image of (**a**) multilayered and (**b**) delaminated Ti_3_C_2_T_x_ obtained via green synthesis.

The morphology of the delaminated Ti_3_C_2_T_x_ material was investigated using high-resolution scanning electron microscopy. Figure 4a shows a multilayered Ti_3_C_2_T_x_ MXene structure with a characteristic accordion-like morphology, typical of stacked Ti_3_C_2_T_x_ layers after selective etching of the MAX phase. Figure 4b displays the morphology after DMSO-assisted delamination, where thinner, exfoliated flakes are visible, indicating a high degree of layer separation.

**Figure 5:**
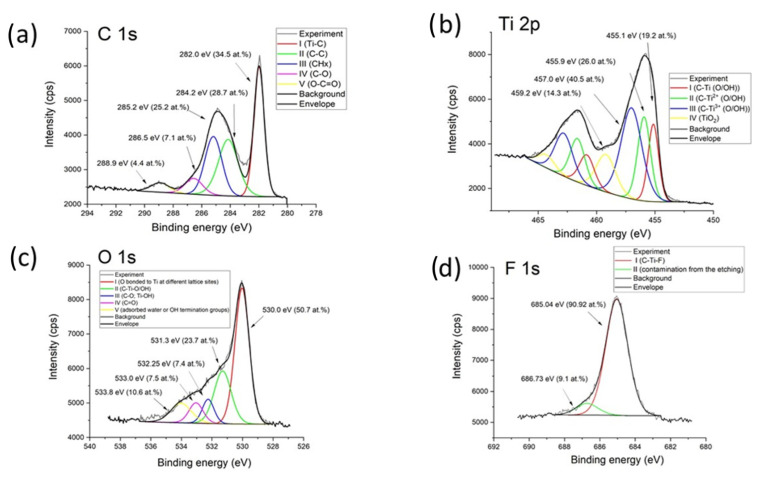
XPS analysis of Ti_3_C_2_T_x_ obtained by a green synthesis method. Numerical fitting of (**a**) C 1s, (**b**) Ti 2p, (**c**) O1s, and (**d**) F1s peaks.

X-ray photoelectron spectroscopy (XPS) is a powerful tool for probing the surface chemistry and bonding states of MXenes. The high-resolution spectra of C 1s, Ti 2p, O 1s, and F 1s provide critical insights into the chemical composition, oxidation states, and functional groups present on the MXene surface, and the measured spectra are given in Figure 5. The C 1s spectrum of MXene typically consists of multiple contributions [79]. The dominant peak at ~282.0–283.0 eV is attributed to Ti-C bonds in the MXene lattice (Ti_3_C_2_ or similar), confirming the presence of carbide carbon.

A second component at ~284.5–285.0 eV corresponds to adventitious carbon (C-C/C-H) from surface contamination, commonly observed in air-exposed samples. A higher binding energy peak (~286–289 eV) may arise from C-O (hydroxyl/ether), C=O (carbonyl), or O-C=O (carboxyl) species, indicating partial oxidation or organic residues. The presence of oxidized carbon species suggests that the Ti_3_C_2_T_x_ surface has undergone some degree of oxidation, possibly due to exposure to ambient conditions or incomplete etching during synthesis. The relative intensities of these peaks can be used to assess the degree of surface functionalization versus contamination. The Ti 2p region is particularly informative for understanding the oxidation state of titanium in Ti_3_C_2_T_x_ [79]. The spectrum is typically deconvoluted into spin-orbit doublets (Ti 2p_3_/_2_ and Ti 2p_1_/_2_, separated by ~5.7 eV). The lowest binding energy doublet (~454.5–455.5 eV for Ti 2p_3_/_2_) corresponds to C-Ti bonds in the Ti_3_C_2_T_x_ core, indicative of Ti in a low oxidation state (Ti^2+^ or Ti^3+^). Peaks at ~456.0–457.5 eV suggest Ti^3+^ (e.g., in Ti_2_O_3_-like environments), which may arise from surface oxidation or defects. The highest binding energy components (~458.0–459.5 eV) are assigned to Ti^4+^ (TiO_2_-like species), confirming partial oxidation of Ti atoms, likely due to surface hydroxylation or exposure to air. The presence of Ti^3+^ and Ti^4+^ states indicates that the Ti_3_C_2_T_x_ surface is not purely Ti-C bonded but contains oxidized titanium species, which can influence its electrochemical and catalytic properties. The O 1s spectrum reflects the diversity of oxygen-containing functional groups on the Ti_3_C_2_T_x_ surface. The main peak at ~530.0–531.0 eV is assigned to Ti-O bonds (lattice oxygen in TiO_2_ or Ti-O-Ti_3_C_2_T_x_), confirming surface oxidation. A second contribution at ~531.5–532.5 eV corresponds to hydroxyl groups (Ti-OH) and/or adsorbed water. A higher binding energy component (~533–534 eV) may indicate C-O or physisorbed water. The relative intensities of these peaks provide insight into the hydration state of the Ti_3_C_2_T_x_. A strong Ti-OH contribution suggests that the material is highly hydroxylated, which is typical for Ti_3_C_2_T_x_ stored under ambient conditions. The F 1s signal is crucial for assessing the presence of residual fluorine from the ammonium bifluoride (NH_4_HF_2_). A peak at ~684.5–685.5 eV is characteristic of Ti-F bonds, confirming that fluorine terminations remain on the surface. A minor contribution at higher binding energy (~687–689 eV) may arise from Al-F (if Al is present from MAX phase impurities). The XPS analysis reveals that the Ti_3_C_2_T_x_ sample consists of a mixed-terminated surface with -O, -OH, and -F groups, along with partial oxidation of titanium and carbon. The presence of TiO_2_-like species suggests that the material has undergone some degree of surface oxidation, which is common for Ti_3_C_2_T_x_ exposed to air.

XRD and Raman analyses (Figure S5) confirmed the formation of single-phase Ti_3_C_2_T_x_, indicating complete removal of the Ti_3_AlC_2_ MAX phase. The sharp, intense XRD peaks reveal high crystallinity, with the strongest peak corresponding to the 002 plane (Figure S5a). Raman spectroscopy was used to compare unexfoliated (multilayer) and delaminated (few-/single-layer) Ti_3_C_2_T_x_ MXene prepared with DMSO (Figure S5b). Unexfoliated samples showed strong low-frequency modes (<300 cm^−1^) from Ti–Ti vibrations and interlayer interactions, along with a dominant A_1g_ mode (200–250 cm^−1^) and broadened E_g_ modes (~400–600 cm^−1^), indicating ordered stacking with some disorder. Delamination suppressed low-frequency modes, shifted and broadened the A_1g_ peak (reflecting weaker interlayer coupling), and enhanced E_g_ intensity, suggesting greater in-plane vibrational freedom. These changes indicate increased surface area, higher defect density, and more accessible active sites, which benefit catalysis and energy storage but may reduce conductivity.

BET analysis showed a large increase in surface area from 8.89 m^2^/g for pristine, multilayer Ti_3_C_2_T_x_ to 230.71 m^2^/g for DMSO-delaminated Ti_3_C_2_T_x_, indicating successful exfoliation into few-/monolayer flakes (Figure S6). The increase results from disrupted interlayer interactions and higher porosity, which can enhance biosensor performance through improved analyte adsorption and electrochemical activity. These values align with literature reports for non-exfoliated (<20 m^2^/g) and delaminated (~200–300 m^2^/g) MXenes.

#### 3.3.2. MoS_2_ Characterization

**Figure 6:**
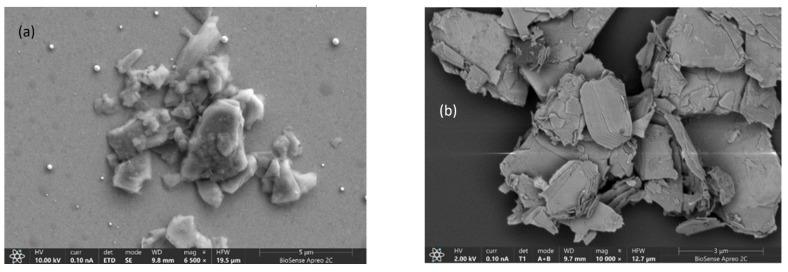
SEM image of MoS_2_ sample after (**a**) 10 min of exfoliation and (**b**) 3h of sonication.

To evaluate the exfoliation efficiency of MoS_2_ under different sonication times, scanning electron microscopy (SEM) was employed. Samples subjected to varying sonication periods were analyzed to assess the impact on structural characteristics and exfoliation degree. SEM imaging provided nanoscale resolution of morphological changes, offering critical insights into the efficacy of sonication as an exfoliation method. Figure 6a shows a scanning electron microscopy (SEM) image of exfoliated MoS_2_ after 10 min of sonication. The image reveals aggregates of exfoliated molybdenum disulfide layers, with clearly discernible flakes of varying sizes. A more detailed examination of the image demonstrates a heterogeneous distribution of exfoliated particles, suggesting that while a 10 min sonication period is sufficient to initiate the exfoliation process, it is inadequate to achieve complete material homogenization.

Figure 6b presents the sample of molybdenum disulfide exfoliated after 3 h of sonication, demonstrating significant progress compared with previous SEM images. The micrograph reveals a higher degree of exfoliation, with clearly visible, thinner, and more uniform material layers. The extended sonication time enabled more efficient separation of MoS_2_ layers, resulting in reduced agglomeration and an increased number of thin flakes. The image shows improved thickness and uniformity of exfoliated layers, indicating a more consistent exfoliation process during prolonged treatment. A bright white line is observed traversing the sample’s central region. This artifact likely originates from intense electron beam exposure, which can induce localized effects such as charging or overheating, leading to the formation of bright traces. These findings confirm the hypothesis that extended sonication time enhances MoS_2_ exfoliation, yielding more homogeneous layer distribution and minimized agglomeration. The results demonstrate the critical role of processing duration in achieving optimal material characteristics for advanced applications.

#### 3.3.3. GO Characterization

**Figure 7:**
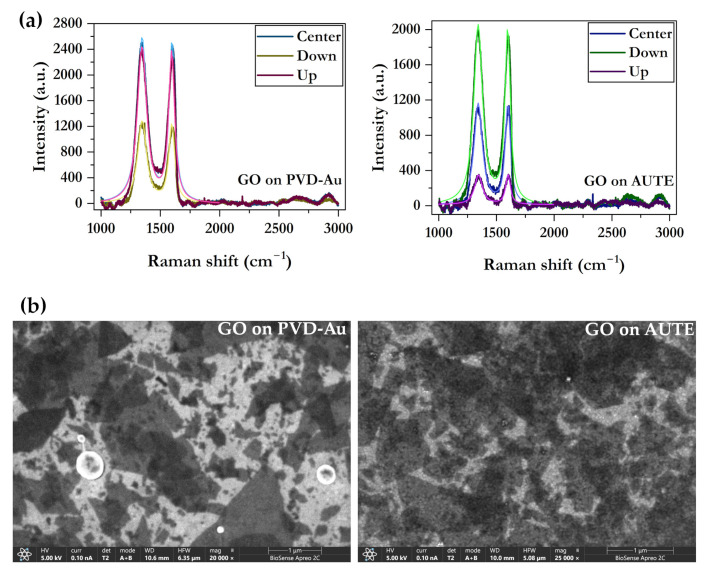
(**a**) Raman spectra of GO on PVD-Au (**left**) and AUTE100 (**right**) electrodes on three WE positions, (**b**) SEM micrographs of GO on gold surface of the PVD-Au (**left**) and AUTE (**right**) electrodes.

Raman spectroscopy reveals the successful deposition of GO onto the modified Au surface on two types of electrodes (Figure 7a) (commercial AUTE100 and in-house gold electrode prepared by PVD deposition). Namely, using the point measurements on different positions of the WE, Raman spectra show strong D and G bands of GO material of similar average intensities (intensity ratio, I_D_/I_G_, 1.048 ± 0.012 and 1.015 ± 0.021 for PVD-Au and AUTE100, respectively), which is typical for GO as it contains large amounts of defects on its surface. Additionally, Raman shifts of the two bands are 1345.4 ± 0.4 cm^−1^ (D) and 1595.55 ± 1.34 cm^−1^ (G) for PVD-Au and 1342.47 ± 1.35 cm^−1^ (D) and 1598.56 ± 1.63 cm^−1^ (G) for the AUTE100 electrode, showing a high similarity in the GO films deposited via drop casting over morphologically different gold surfaces.

SEM micrographs give the GO film morphology and the assembly of GO sheets deposited via a drop-casting method described in Materials and Methods. As shown in Figure 7b, GO flakes are of different lateral dimensions dispersed randomly over the gold surface, mostly forming a monolayer film with a slight flake overlap. The obtained monolayer film of GO is a consequence of electrostatically anchored negatively charged GO sheets to the positively charged cysteamine-modified gold. Larger flakes are approximately 1 μm in diameter, while smaller flakes are down to ~100 nm.

### 3.4. Functionalization of the Electrodes with Various Nanomaterials and DNA Probe Immobilization

**Figure 8:**
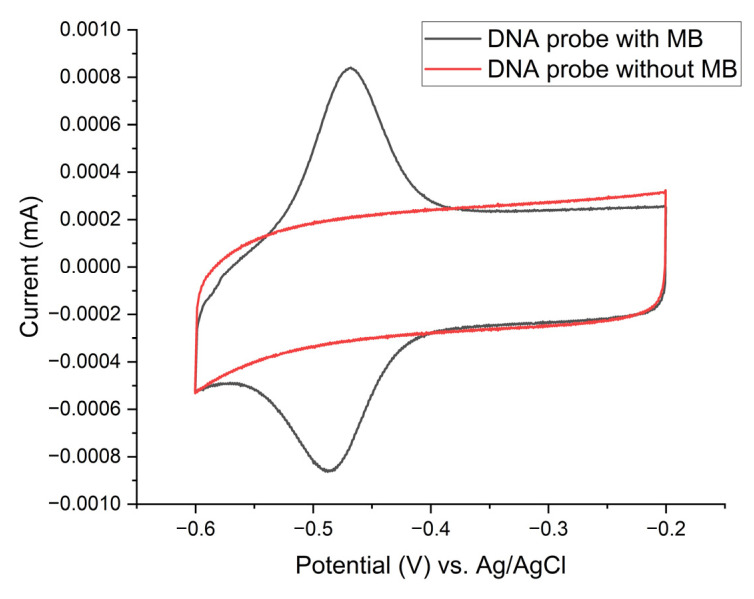
Cyclic-voltammogram comparison for laser-patterned gold-leaf electrodes bearing linear oligonucleotide DNA probes either labelled or unlabeled with methylene blue–MB (surface density of 750 pmol/cm^2^, P-35S region). Voltammograms were recorded in 1 × PBS at 100 mV/s within a potential window of −0.60 to −0.20 V vs. a saturated Ag/AgCl reference electrode.

The presence of MB on an oligonucleotide DNA probe immobilized on the gold electrode surface is illustrated in Figure 8. The MB-tagged probes exhibit a well-defined redox peak centered at around −0.48 V, whereas no corresponding feature is present for probes lacking MB, confirming the successful immobilization of the MB-labeled oligonucleotide DNA probe on the electrode surface.

#### 3.4.1. Gold Electrode Comparison in DNA Detection, Repeatability, Real Sample Detection, and Calibration

The morphology of the gold electrode surface and the specific production methods used have a significant impact on both catalytic activity and the effective active surface area. Variations in surface roughness, nanostructuring, and patterning can dramatically alter the number and accessibility of active catalytic sites. Features such as nanopores, nanoleaves, or roughened surfaces increase the available area for reactions, enhancing overall catalytic efficiency. Production techniques such as electrodeposition, laser patterning, or chemical etching determine the final surface architecture. These methods can create high-surface-area structures that promote better electron transfer and improve the electrode’s catalytic properties.

**Figure 9:**
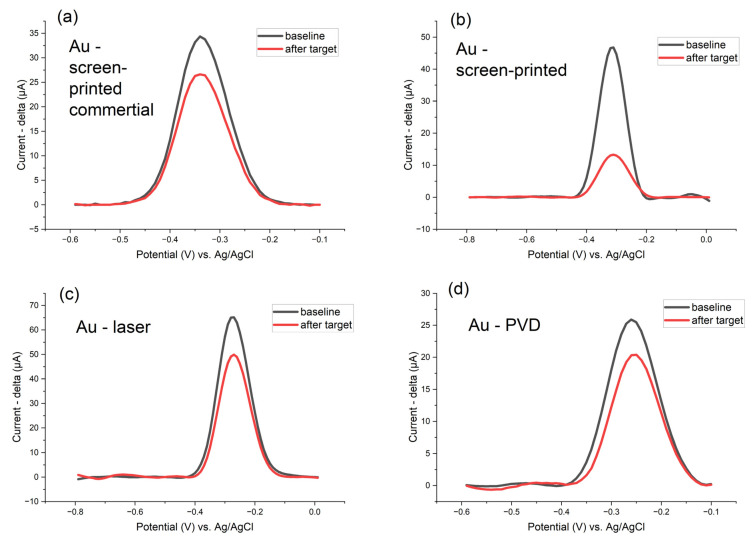
Detection of target DNA molecules (amplification products of LAMP reaction) by linear oligonucleotide DNA probes on four different types of gold electrodes—(**a**) commercial screen-printed gold (Zensor AUTE100), (**b**) screen-printed sintered gold at 800 °C, (**c**) laser-patterned gold leaf, and (**d**) PVD gold. In each case, linear DNA probes (surface density of 750 pmol/cm^2^, P-35S region) bearing methylene blue (MB) were employed. Baseline responses (black line) were recorded in 1 × PBS by square-wave voltammetry (pulse amplitude 75 mV, pulse width 2 ms, step potential 10 mV, potential window −0.60 to −0.10 V vs. saturated Ag/AgCl). Red lines show the signals after 1 h hybridization with 30 min LAMP products (template gBlock concentration 10^−3^ ng/µL diluted 100 times in 1 × PBS), measured under identical conditions.

The comparison of DNA detection on four different types of gold electrodes—PVD gold, commercial screen-printed gold (Zensor AUTE100), laser-patterned gold leaf, and screen-printed sintered gold at 800 °C—is shown in Figure 9. Laser-patterned gold electrodes showed the highest currents, but screen-printed homemade gold electrodes gave the highest sensitivity with a peak decrease several times.

**Figure 10:**
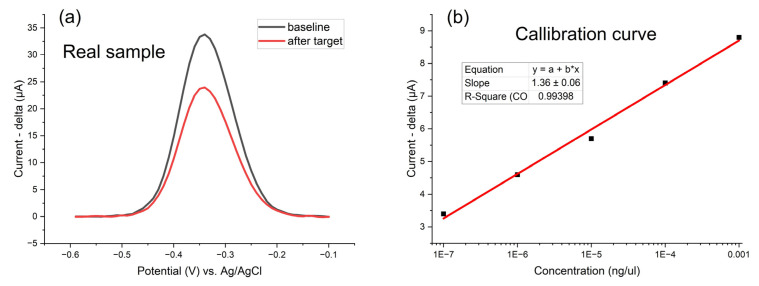
(**a**) The detection of target P-35S region after LAMP reaction using rapeseed leaf DNA spiked with a P-35S gBlock; (**b**) Calibration curve for the electrochemical detection of LAMP products produced using P-35S gBlock template at concentrations ranging from 10^−3^ to 10^−7^ ng/µL, diluted in 1× PBS, using commercial screen-printed gold electrodes (Zensor AUTE100); linear oligonucleotide DNA probes (surface density of 750 pmol/cm^2^, P-35S region) carrying methylene blue (MB) were employed; square-wave voltammetry (pulse amplitude 75 mV, pulse width 2 ms, step potential 10 mV, potential window −0.60 to −0.10 V vs. saturated Ag/AgCl); signals are obtained after 1 h hybridization with 30 min LAMP reaction products; for each starting gBlock concentration, the decrease in peak is shown in μA.

Reliability of the detection using SWV is shown in Figures S7–S9, highlighting the repeatability of both the baseline and the DNA detection signal. The detection of the target P-35S region after the LAMP reaction using rapeseed leaf DNA spiked with a P-35S gBlock (simulating a real GM sample) is presented in Figure 10a, representing real sample detection. A calibration curve (Figure 10b) is also included to illustrate the trend of target detection, LOD (template gBlock concentrations ~10^−7^ ng/µL), and the sensitivity of the method.

P-35S LAMP products produced using rapeseed leaf DNA spiked with a P-35S gBlock (simulates a real GM sample) were used for the validation of the electrochemical detection. The decrease in peak was very similar to that from LAMP products of clean gBlock targets.

#### 3.4.2. Gold Nanoparticles Electrodeposition on Four Types of Au Electrodes and Carbon Electrodes—DNA Detection Comparison

Gold nanoparticles significantly enhance the sensitivity of DNA electrochemical sensors by increasing probe density, improving electron transfer, and enabling powerful signal amplification strategies. These enhancements allow for ultra-low detection limits, robust discrimination of single-base mismatches, and reliable detection in complex samples, making AuNPs a cornerstone in the development of next-generation DNA biosensors [80,81]. Therefore, we have utilized commercial Au nanoparticles to improve detection performance. The morphology of Au nanoparticles was investigated by the SEM technique. In Figure S10, the SEM image of dispersed Au nanoparticles is shown, revealing that their size ranges from 60 nm to 80 nm and that there are no observable agglomerates or other types of particles.

**Figure 11:**
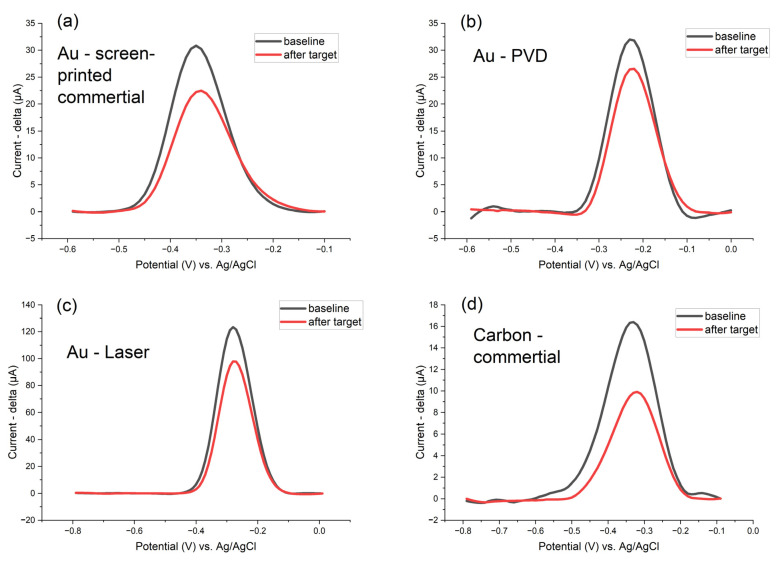
Detection of target DNA molecules (amplification products of LAMP reaction) by linear oligonucleotide DNA probes on four gold-nanoparticle-decorated electrode platforms: (**a**) commercial screen-printed gold (Zensor AUTE100), (**b**) PVD-coated gold, (**c**) laser-patterned gold leaf, and (**d**) a carbon electrode (Zensor TE100) modified with electrodeposited gold nanoparticles (AuNPs). In every case, linear oligonucleotide DNA probes (surface density of 750 pmol/cm^2^, P-35S region) carrying methylene blue (MB) were employed. Baseline responses (black traces) were recorded in 1× PBS by square-wave voltammetry (pulse amplitude 75 mV, pulse width 2 ms, step potential 10 mV, potential window −0.60 to −0.10 V vs. saturated Ag/AgCl). Red traces depict the signals after 1 h hybridization with 30 min LAMP reaction products (template gBlock concentration 10^−3^ ng/µL diluted 1:100 in 1× PBS), measured under the same conditions.

Electrodeposition of AuNPs was performed on four distinct gold-nanoparticle-decorated electrode platforms (Figure 11) to compare their responses to target DNA detection, that is, the amplification products of the LAMP reaction. Carbon electrodes decorated with AuNPs demonstrated the highest sensitivity, evidenced by an almost 50% reduction in signal upon target DNA detection. The significant signal decrease indicates enhanced hybridization efficiency and effective electron transfer, making it particularly suitable for ultrasensitive DNA sensing. Gold laser-patterned electrodes with AuNPs exhibited the strongest SWV signal among all tested platforms. The superior signal strength is attributed to the exceptionally high surface area of the laser-patterned gold “leaves”, which is further amplified by the presence of AuNPs. This structural advantage maximizes probe loading (Figure S11) and electron transfer, resulting in robust detection signals.

Buffer concentration can significantly impact the sensitivity of DNA detection signals by inducing conformational changes in both oligonucleotide DNA probes and target DNA molecules, influenced by the ionic strength of the solution. Figure S12 illustrates the effect of PBS concentration on LAMP reaction product detection. The detection signal for target DNA molecules (amplification products of LAMP reaction) is significantly stronger in 1× concentrated PBS, as evidenced by a several-fold increase in the measured current. Additionally, the sensitivity, indicated by the peak drop, shows a slight improvement under these conditions.

### 3.5. Electrochemical Detection of Target DNA Molecules

#### 3.5.1. Optimal DNA Target Concentration for Sensitive Electrochemical Detection

Detection of the optimal target DNA concentration for achieving the most sensitive detection in DNA biosensors and hybridization assays depends on the sensor platform, probe density, detection method, and the complexity of the DNA amplification products. This means that the optimal target concentration for achieving the highest sensitivity effectively defines the sensor’s detection range and overall performance. Recent studies consistently demonstrate that the most sensitive electrochemical DNA detection using LAMP amplification is achieved at extremely low target concentrations, often in the attomolar (aM, 10^−18^ M) to low femtomolar (fM, 10^−15^ M) range [82,83,84].

#### 3.5.2. Comparison of the Electrochemical Techniques for DNA Target Detection

**Figure 12:**
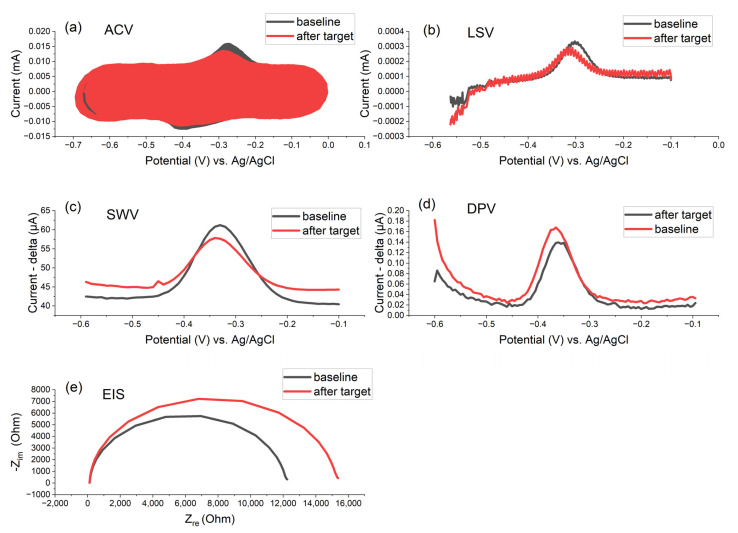
Comparison of RAW data of the same electrochemical system with various electrochemical methods: (**a**) ACV, (**b**) LSV, (**c**) SWV, (**d**) DPV, and (**e**) EIS; P-35S oligonucleotide DNA probes (surface density of the probes 750 pmol/cm^2^ with MB) immobilized on Zensor AUTE100 commercial gold electrodes–in 1× PBS (10 mM K_3_/K_4_(Fe(CN)_6_) 1:1, in 100 mM KCl, for EIS measurement) after 1 h of DNA hybridization; square wave voltammetry (pulse amplitude 75 mV, pulse width 2 ms, step potential 10 mV, potential window −0.60 to −0.10 V vs. saturated Ag/AgCl); EIS parameters 1−100000 Hz and 10 mV amplitude; the detection of target DNA molecules (30 min LAMP products made with 10^−3^ ng/ul of template gBlock, 100× diluted in 1× PBS after the LAMP reaction).

Electrochemical DNA biosensors employ various techniques to detect DNA hybridization and quantify target sequences, such as Electrochemical Impedance Spectroscopy (EIS), Differential Pulse Voltammetry (DPV), Square Wave Voltammetry (SWV), Linear Sweep Voltammetry (LSV), and Alternating Current Voltammetry (ACV), each offering distinct signal amplification characteristics and invasiveness towards sensitive biomolecules (Figure 12). The ability to amplify a signal directly impacts sensitivity, detection limits, and suitability for low-abundance DNA targets.

Pulse techniques (DPV, SWV) provide the strongest signal amplification and background suppression, making them ideal for ultra-sensitive DNA detection [85]. EIS achieves high amplification when paired with advanced surface engineering but relies on indirect signal enhancement. LSV and ACV offer moderate amplification; their sensitivity can be improved with surface modifications or harmonic analysis, but they generally lag behind DPV and SWV [86,87]. The choice of technique and amplification strategy should match the required detection limit, sample complexity, and assay format [45,88].

All electrochemical techniques shown in Figure 12 exhibit a decrease in the MB signal following the detection of the target DNA molecule (amplification product of the LAMP reaction). These techniques vary in response speed—some methods provide faster detection, while others operate more slowly; invasiveness—the extent to which each technique affects sensitive biomolecules differs, with certain methods being gentler and others potentially more disruptive; sensitivity—detection limits and the ability to discern low concentrations of DNA targets can vary significantly between techniques; and repeatability and baseline stability—the consistency of results and the stability of the baseline signal differ, impacting the reliability of each method.

The choice of the most suitable electrochemical technique depends on the detection mechanism, the configuration of the overall system, and the required detection range. Each method offers specific advantages depending on these factors. It is crucial to carefully optimize the setup parameters for each electrochemical technique, as these settings can greatly influence the quality and reliability of the output signal. In the comparison presented in Figure 12, SWV demonstrates the most stable baseline and delivers the highest sensitivity among the evaluated methods.

Square wave voltammetry stands out due to its combination of high sensitivity, rapid analysis, strong signal amplification, and robust background suppression. These features make SWV particularly advantageous for ultrasensitive, high-throughput, and multiplexed DNA sensing applications, both in research and point-of-care diagnostics [89,90].

#### 3.5.3. Selectivity of the Detection and Various DNA Probe Testing

**Figure 13:**
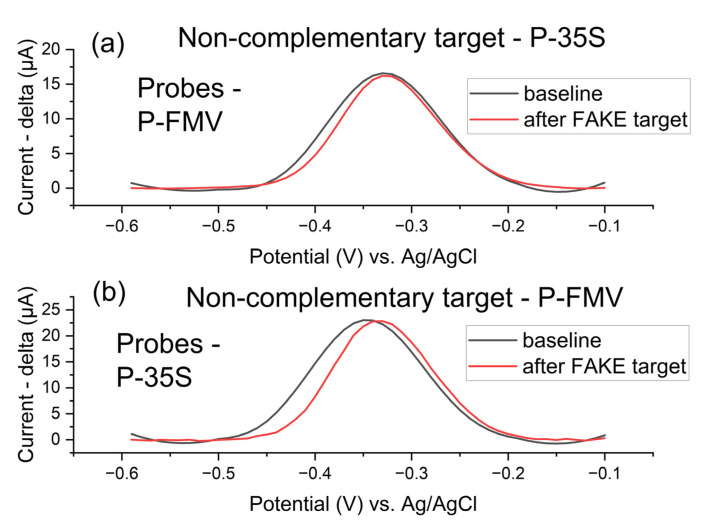
P-FMV (**a**) and P-35S (**b**) oligonucleotide DNA probes (surface density of the probes 750 pmol/cm^2^ with MB) immobilised on Zensor AUTE100 commercial electrodes–comparison of the selectivity of target detection in 1× PBS after 1 h of the potential DNA hybridization detection (non-complementary detection of target P-35S region was performed with P-FMV oligonucleotide DNA probes, and non-complementary detection of target P-FMV region with P-35S oligonucleotide DNA probes); square wave voltammetry (pulse amplitude 75 mV, pulse width 2 ms, step potential 10 mV, potential window −0.60 to −0.10 V vs. saturated Ag/AgCl); the detection of target DNA molecules (30 min LAMP products made with 10^−3^ ng/ul of template gBlock, 100× diluted in 1× PBS after the LAMP reaction).

Selectivity testing was performed for both target DNA regions, P-35S and P-FMV (surface density 1500 pmol/cm^2^), immobilized on commercial Zensor AUTE100 electrodes (Figure 13). The DNA non-complementary detection of the target P-35S region was tested with P-FMV oligonucleotide DNA probes, and the non-complementary detection of the target P-FMV region was tested with P-35S oligonucleotide DNA probes.

**Figure 14:**
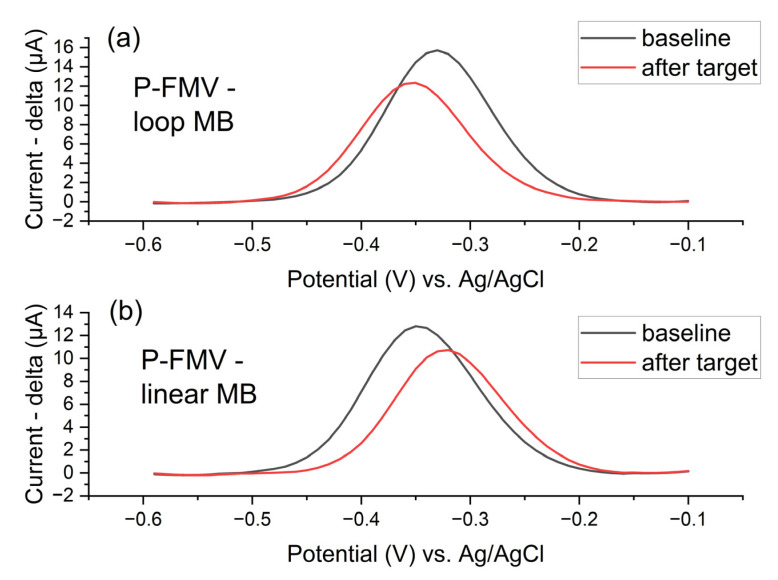
P-FMV oligonucleotide DNA probes (surface density of the probes 750 pmol/cm^2^ with MB) immobilised on Zensor AUTE100 commercial electrodes–comparative influence of oligonucleotide DNA probe conformation ((**a**) loop MB and (**b**) linear MB) on the detection mechanism during target recognition in 1× PBS after 1 h of the DNA hybridization detection; SWV (pulse amplitude 75 mV, pulse width 2 ms, step potential 10 mV, potential window −0.60 to −0.10 V vs. saturated Ag/AgCl); the detection of target DNA molecules (30 min LAMP products made with 10^−3^ ng/ul of template gBlock, 100× diluted in 1× PBS after the LAMP reaction).

A comparison of target DNA detection using linear and loop DNA probes for the P-FMV region is presented in Figure 14. Loop DNA probes exhibit slightly greater sensitivity than linear probes, as indicated by a higher peak drop (red line).

**Figure 15:**
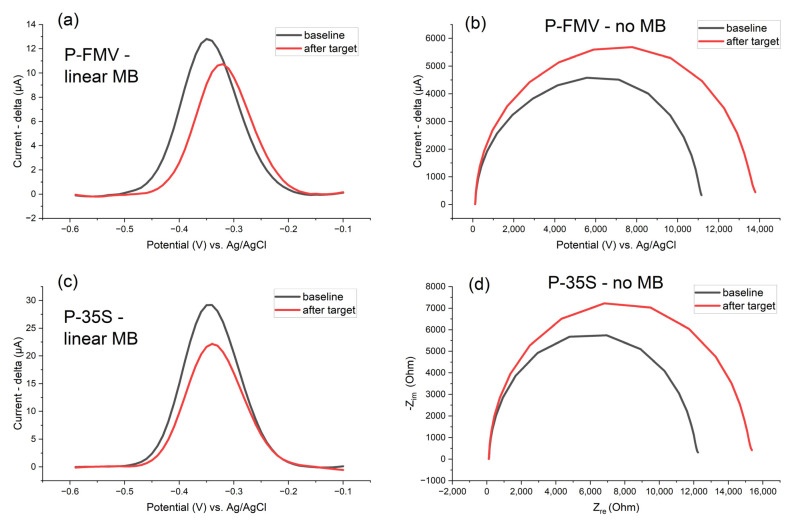
P-FMV and P-35S linear oligonucleotide probes with methylene blue–MB (**a**,**c**) (surface density of the probes 750 pmol/cm^2^ with) immobilised on Zensor AUTE100 commercial electrodes–sensitivity comparison (performance of the designed oligonucleotide DNA probes) for both detection target regions in 1× PBS ((**b**,**d**) 10 mM K_3_/K_4_(Fe(CN)_6_) 1:1, in 100 mM KCl, for EIS experiments with no MB probes) after 1 h of the DNA hybridization detection; SWV (pulse amplitude 75 mV, pulse width 2 ms, step potential 10 mV, potential window −0.60 to −0.10 V vs. saturated Ag/AgCl); EIS parameters 1–100,000 Hz with the amplitude of 10 mV; the detection of target DNA molecules (30 min LAMP products made with 10^−3^ ng/ul of template gBlock, 100× diluted in 1× PBS after the LAMP reaction).

The design quality of the oligonucleotide DNA probes targeting the P-FMV and P-35S regions is demonstrated in Figure 15. Both probes exhibited satisfactory detection sensitivity, as indicated by the detection peak drop in SWV (red line) and higher semicircle difference in EIS Nyquist diagrams. However, the oligonucleotide DNA probe for the P-35S region produced a slightly stronger signal and higher sensitivity (higher peak and semicircle difference) compared with the P-FMV region.

#### 3.5.4. Comparison of Various Nanomaterials in Detection Sensitivity

**Figure 16:**
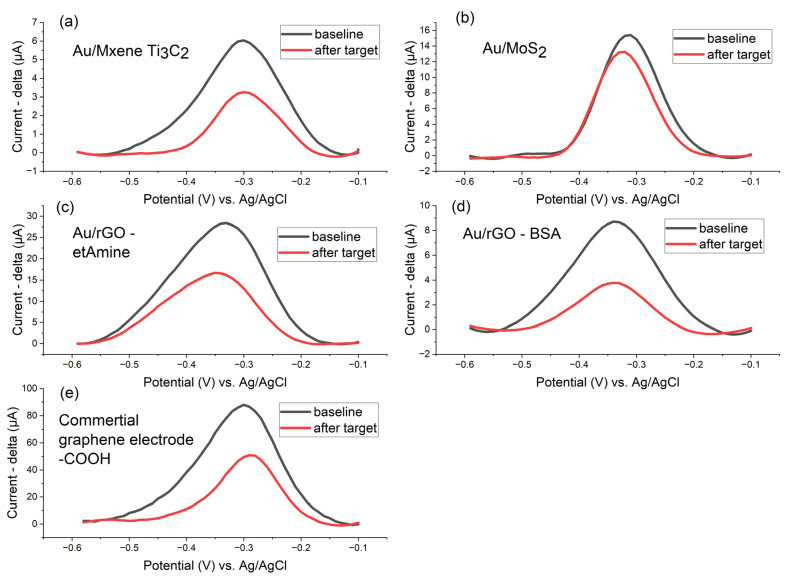
The detection of target DNA molecules by linear oligonucleotide DNA probes on three distinct two-dimensional nanomaterials: (**a**) MXene (Ti_3_C_2_T_x_), (**b**) molybdenum disulfide (MoS_2_), rGO with (**c**) ethanolamine and (**d**) BSA, and (**e**) commercial graphene electrode -COOH-functionalized; linear oligonucleotide DNA probes with MB (surface density of 750 pmol/cm^2^, P-35S region) were used; baseline (black lines) is recorded in 1 × PBS (square wave voltammetry, pulse amplitude 75 mV, pulse width 2 ms, step potential 10 mV, potential window −0.60 to −0.10 V vs. saturated Ag/AgCl), and the detection of target DNA molecules (30 min LAMP products made with 10^−3^ ng/ul of template gBlock, 100× diluted after the reaction in 1× PBS) after 1 h of hybridization, again in 1× PBS (red lines).

We developed an electrochemical biosensor to detect amplified DNA (LAMP products from 10^−3^ ng/μL gBlock template) using methylene blue-tagged DNA probes (750 pmol/cm^2^) targeting the P-35S sequence. The detection mechanism relies on hybridization-induced displacement of the redox reporter, causing measurable current reduction in square-wave voltammetry (53% signal decrease for optimal Au/rGO electrodes with BSA blocking). While rGO-modified electrodes showed the highest sensitivity, emerging nanomaterials like MXenes (46% signal decrease) and MoS_2_ (14%) demonstrated promising performance, suggesting their potential for future biosensor development, as depicted in Figure 16.

While previous studies have made significant strides in nucleic acid detection, our work distinguishes itself through a combination of innovative material science, cost-efficient and green synthesis methods, and comprehensive performance benchmarking. The study described in research published by Zhao et al. [91] demonstrates high-throughput fluorescence-based SARS-CoV-2 detection using rGO, achieving remarkable sensitivity. However, reliance on optical readouts inherently limits portability, a challenge that our work directly addresses by employing electrochemical detection.

The research reported by Martin et. al. [92] demonstrates impressive single-copy DNA detection sensitivity using electrochemical LAMP. While their work achieves remarkable performance with intercalating redox reporters, it examines only a single electrode type without exploring alternative nanomaterials. In contrast, our study provides the first comprehensive comparison of three distinct 2D nanomaterials (rGO, Ti_3_C_2_T_x_, and MoS_2_) across multiple electrode architectures while also introducing an environmentally friendly synthesis route for Ti_3_C_2_T_x_ that eliminates hazardous HF/HCl reagents. The work presented by Yu et al. [93] develops an ultrasensitive biosensor for cancer DNA detection using AuNPs@Ti_3_C_2_ nanocomposites. Although innovative, their platform focuses exclusively on KRAS G12D detection without investigating other nanomaterials or electrode configurations. Our approach offers broader applicability through systematic evaluation of multiple nanomaterial classes while specifically addressing the unmet need for GMO screening tools in food safety applications. The ratiometric LAMP sensor described by Fu et al. [94] achieves excellent sensitivity for pathogen detection using AuNPs@MoS_2_ and dual-signal readouts. However, their reliance on complex host–guest chemistry (Fc/β-CD) may limit practical implementation. Our design simplifies the sensing architecture while maintaining high performance through optimized nanomaterial interfaces and avoids proprietary reagents, making it more suitable for field-deployable GMO monitoring applications.

This work establishes a robust framework for nucleic acid detection that balances sensitivity with practical applicability, highlighting how material selection critically influences biosensor performance.

## 4. Conclusions

The presented research showcased the development of advanced electrochemical biosensing platforms for the detection of transgenic regulatory elements by integrating LAMP amplification with 2D nanomaterial-modified electrodes. The materials research was guided by the green approach, where an innovative synthesis method was applied for obtaining 2D nanomaterial nanosheets and their further functionalization. The synergistic properties of rGO, Ti_3_C_2_T_x_ and MoS_2_, notably their high surface area and exceptional conductivity, enabled superior signal transduction and detection sensitivity. Through systematic evaluation of custom-fabricated electrodes (laser-patterned Au leaf, PVD-deposited Au, and screen-printed Au), alongside commercial counterparts, we elucidated the critical impact of fabrication methodology and material composition on biosensor performance, including sensitivity, reproducibility, and detection efficiency. The optimized platform demonstrates high specificity, rapid response, and robustness, making it a viable candidate for on-site GMO screening. By combining sustainable nanomaterial synthesis with precision biosensor engineering, this work advances electrochemical nucleic acid detection for agricultural biotechnology and food safety monitoring. To enhance the performance of rGO, Ti_3_C_2_T_x_, and MoS_2_, future work should optimize their synthesis for higher conductivity and fewer defects. Doping rGO, tuning Ti_3_C_2_T_x_ surface groups, and modifying MoS_2_ oxidation states could improve electron transfer and catalytic activity. Fine-tuning nanocomposite ratios and electrode patterning may further boost sensitivity and speed while reducing costs, enabling more efficient on-site detection. Future efforts will prioritize real sample on-field calibration, validation, and miniaturization to enable portable, real-world deployment.

## Figures and Tables

**Table 1:** Modifications of oligonucleotide probes used in this study.

Thiol + methylene blue (MB) modified linear oligonucleotide probe for electrochemistry	
P-35S	/5ThioMC6-D/GAAGACGTTCCAACCACGTC/3MeBlN/
P-FMV	/5ThioMC6-D/GTGCTGGAACAGTAGTTTACTTTGATTG/3MeBlN/
Thiol + MB-modified stem-loop oligonucleotide probe for electrochemistry modifications	
P-FMV SLP	/5ThioMC6-D/CCGTGTGCTGGAACAGTAGTTTACTTTGATTGACGG/3MeBlN/
Thiol-modified linear oligonucleotide probe for electrochemistry	
P-35S	/5ThioMC6-D/GAAGACGTTCCAACCACGTC-3’
P-FMV	/5ThioMC6-D/GTGCTGGAACAGTAGTTTACTTTGATTG-3’
Amino + MB modified linear oligonucleotide probe for electrochemistry	
P-FMV	/5AmMC6/GTGCTGGAACAGTAGTTTACTTTGATTG/3ATTO MB2/

## Data Availability

Data is contained within the article or Supplementary Material.

## Supplementary Materials

The following supporting information can be downloaded at https://www.mdpi.com/article/10.3390/bios15090584/s1, Supplementary Data 1: LAMP assay validation and oligonucleotide DNA probe design; Supplementary Data 2: Nanomaterials characterization; Supplementary Data 3: Electrochemical detection. References [13,60,61,95,96,97,98,99,100] are cited in the Supplementary Materials.
