# Supplementary material for: Advanced Nanomaterial-Based Electrochemical Biosensing of Loop-Mediated Isothermal Amplification Products
Source: Biosensors (Basel). 2025 Sep 5;15(9):584. doi: 10.3390/bios15090584 (PMC12467743; doi:10.3390/bios15090584)
Supplement: Supplementary file 1 [file biosensors-15-00584-s001.zip › biosensors-3786110-supplementary.pdf]

biosensors

MDPI

Supplementary Materials

Advanced Nanomaterial-Based Electrochemical Biosensing of Loop-Mediated Isothermal Amplification Products

Ana Kuprešanin [1], Marija Pavlović [1], Ljiljana Šašić Zorić [1], Milinko Perić [2], Stefan Jarić [1], Teodora Knežić [1], Ljiljana Janjušević [1], Zorica Novaković [1], Marko Radović [1], Mila Djisalov [1], Nikola Kanas [1], Jovana Paskaš [1] and Zoran Pavlović [1,*]

[1] BioSense Institute, Research and Development Institute for Information Technologies in Biosystems, University of Novi Sad, Dr Zorana Đinđića 1, 21000 Novi Sad, Serbia; ana.kupresanin@biosense.rs (A.K.); ljsasic@biosense.rs (L.Š.Z.)
[2] Institute of Field and Vegetable Crops, Maksima Gorkog 30, 21000 Novi Sad, Serbia
* Correspondence: zoran.pavlovic@biosense.rs

Supplementary Data 1: LAMP Assay Validation and Oligonucleotide DNA Probe Design

Table S1. Data on gBlocks, primers and oligonucleotide probes used.

Type	Name	Sequence (5′-3′)	Source
gBlock	P-35S gBlock	CTACAAATGCCATCATTGCGATAAAGGAAAGGCCATCGTTGAA-GATGCCTCTGCCGACAG-TGGTCCCAAAGATGGACCCCCACCCACGAGGAGCATCGTGGAAAAA GAAGACGTTCCAACCACGTCTTCAAAGCAAGTGGATTGATGTGA-TATCTCCACTGAC-GTAAGGGATGACGCACAATCCCACTATCCTTCGCAAGACCCTTCCTC TATATAAGGAAGTTCATTT	GenBank acc. no. V00141
LAMP primers	P-35S F3	AGGAAGGGTCTTGCG	[60]
	P-35S B3	ATAAAGGAAAGGCCATCG	
	P-35S FIP	GTCTTCAAAGCAAGTGGTTTTGGATAGTGGGATTGTGCG	
	P-35S BIP	TTCCACGAT GCTCCTCGTTTTCCTCTGCCGACAGTGG	
	P-35S LF	TCCACTGACGTAAGGG	
	P-35S LB	GGGGTCCATCTTTGGG	
Oligonucleotide probe	P-35S probe	GAAGACGTTCCAACCACGTC	New design
gBlock	P-FMV gBlock	CTCAAAGGTTTGTAAGGAAGAATTCTCAGTCCAAA-GCCTCAACAAGGTCAGGGTACAGAG-TCTCCAAACCATTAGCCAAAAGCTACAGGAGATCAATGAAGAATCT TCAATCAAAGTAAACTACTGTTCCAGCACATGCATCATGGTCAG-TAAGTTTCAGAAAAAGA-CATCCACCGAAGACTTAAAGTTAGTGGGCATCTTTGAAAGTAATCTT GTCAACATCGAGCAGCTGGCTTGTGGGGACCAGA-CAAAAAAGGAATGGTGCAGAATTGTTAGGCGCACCTACCAAAAGCA	GenBank acc. no. NC_003554
LAMP primers	P-FMV F3	AACAATTCTGCACCATTCCT	[13]
	P-FMV B3	AATTCTCAGTCCAAAGCCTC	
	P-FMV FIP	TGCATCATGGTCAGTAAGTTTCAGATGCTCGATGTTGACAAGATT	
	P-FMV BIP	TGTGCTGGAACAGTAGTTTACTTTGAAGGTCAGGGTACAGAGTC	
	P-FMV LF	AAGACATCCACCGAAGACTTAA	
	P-FMV LB	AGATTCTTCATTGATCTCCTGTAGC	
Oligonucleotide probe	P-FMV probe	GTGCTGGAACAGTAGTTTACTTTGATTG	New design

 https://doi.org/10.3390/bios15090584

gBlock	T-nos gBlock	AGGAGTGCGTCGAAGCAGATCGTTCAAACATTTGGCAA-TAAAGTTTCTTAA-GATTGAATCCTGTTGCCGGTCTTGCGATGATTATCATATAATTTCTGTTGAATTACGTTAAGCATGTAATAATTAACATGTAATGCATGAC-GTTATTTATGA-GATGGGTTTTTATGATTAGAGTCCCGCAATTATACATTTAATACGCGATAGAAAACAAAATATAGCGCGCAAACTAGGA-TAAATTATCGCGCGCGGTGTCATCTATGTTAC	GenBank acc. no. V00087
LAMP pri-mers	T-nos F3	CGCGATAATTTATCCTAGTTTG	[61]
	T-nos B3	CGTTCAAACATTTGGCAAT	
	T-nos FIP	GCATGACGTTATTTATGAGATGGGT-TTT-CGCTATATTTTGTTTTC-TATCGCG	
	T-nos BIP	CATGCTTAACGTAATTCAACAG-TTT-TTGAATCCTGTTGCCGGTC	
	T-nos LF	GATTAGAGTCCCGCAATTATAC	
	T-nos LB	AAATTATATGATAATCATCGCAA	

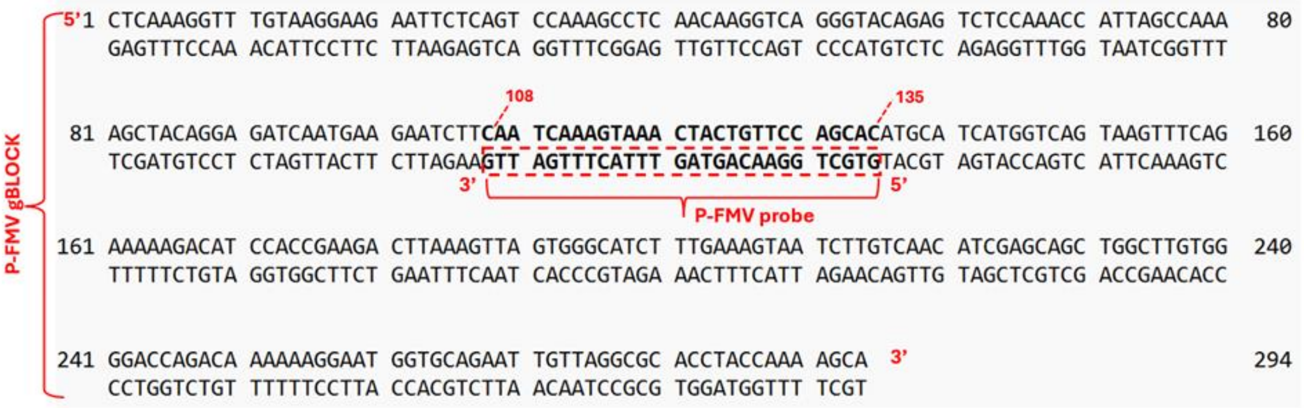

Figure S1. Illustration of the specific probe (P-FMV probe)-binding site within the target gBlock (P-FMV gBLOCK). The P-FMV probe sequence (5′→3′) is shown directly aligned to its complementary region in the target sequence, demonstrating specific binding and probe–target complementarity.

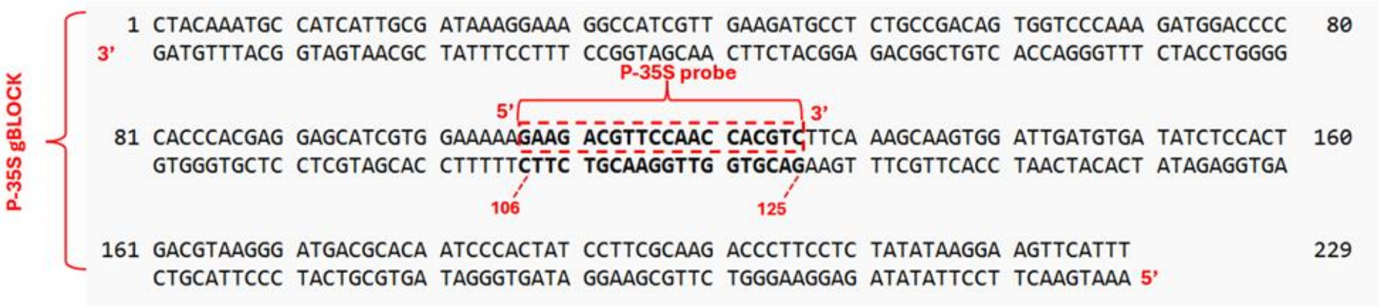

Figure S2. Illustration of the specific probe (P-35S probe)-binding site within the target gBlock (P-35S gBLOCK). The P-35S probe sequence (5′→3′) is shown directly aligned to its complementary region in the target sequence, demonstrating specific binding and probe–target complementarity.

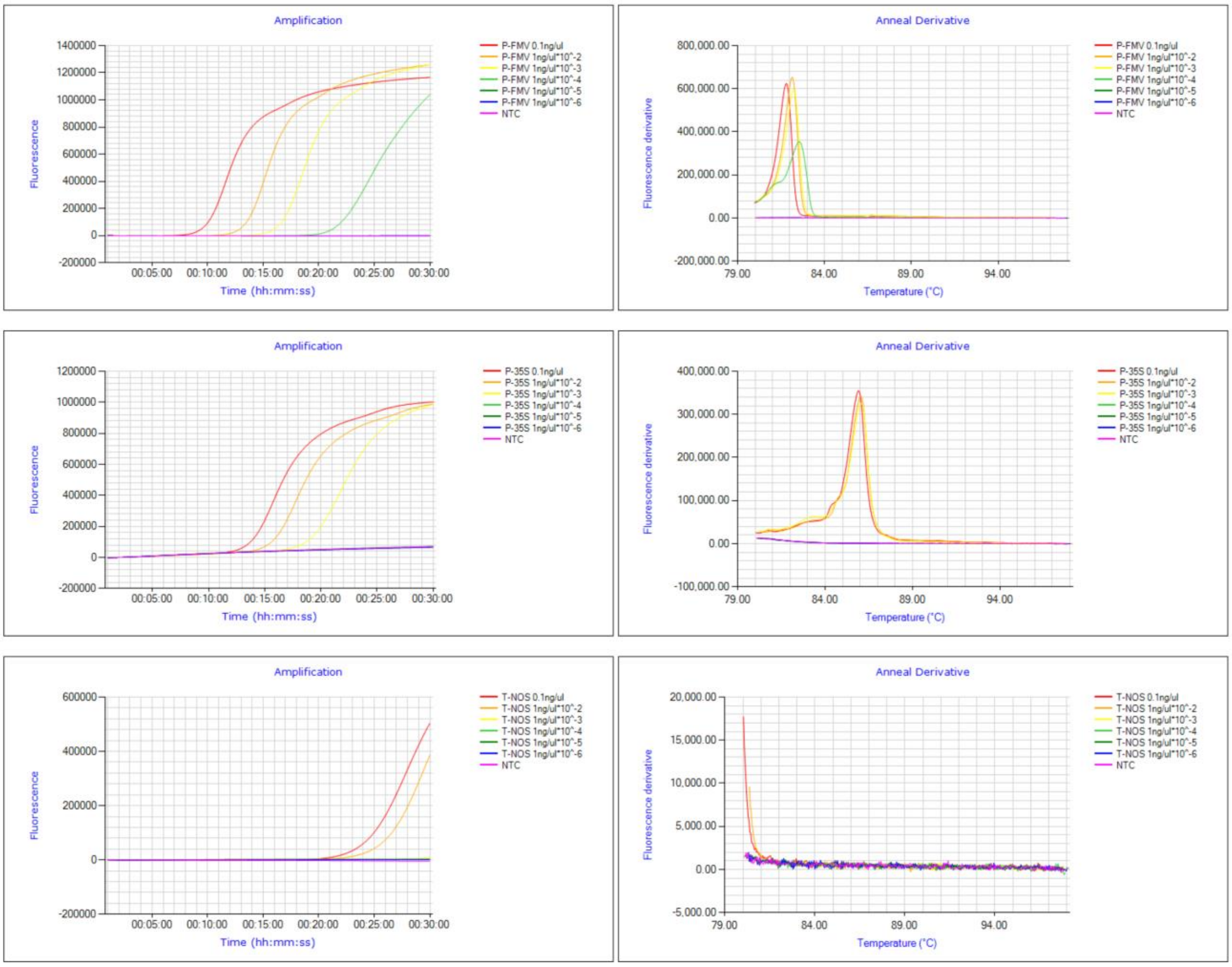

Figure S3. Estimation of the limit of detection (LOD) of regulatory elements of transgenic construct (P-35S, P-FMV, and T-nos) using artificial target sequences (gBlocks), amplification curves, and melting profiles from Real Time LAMP reactions. The legend indicates color-coded reactions.

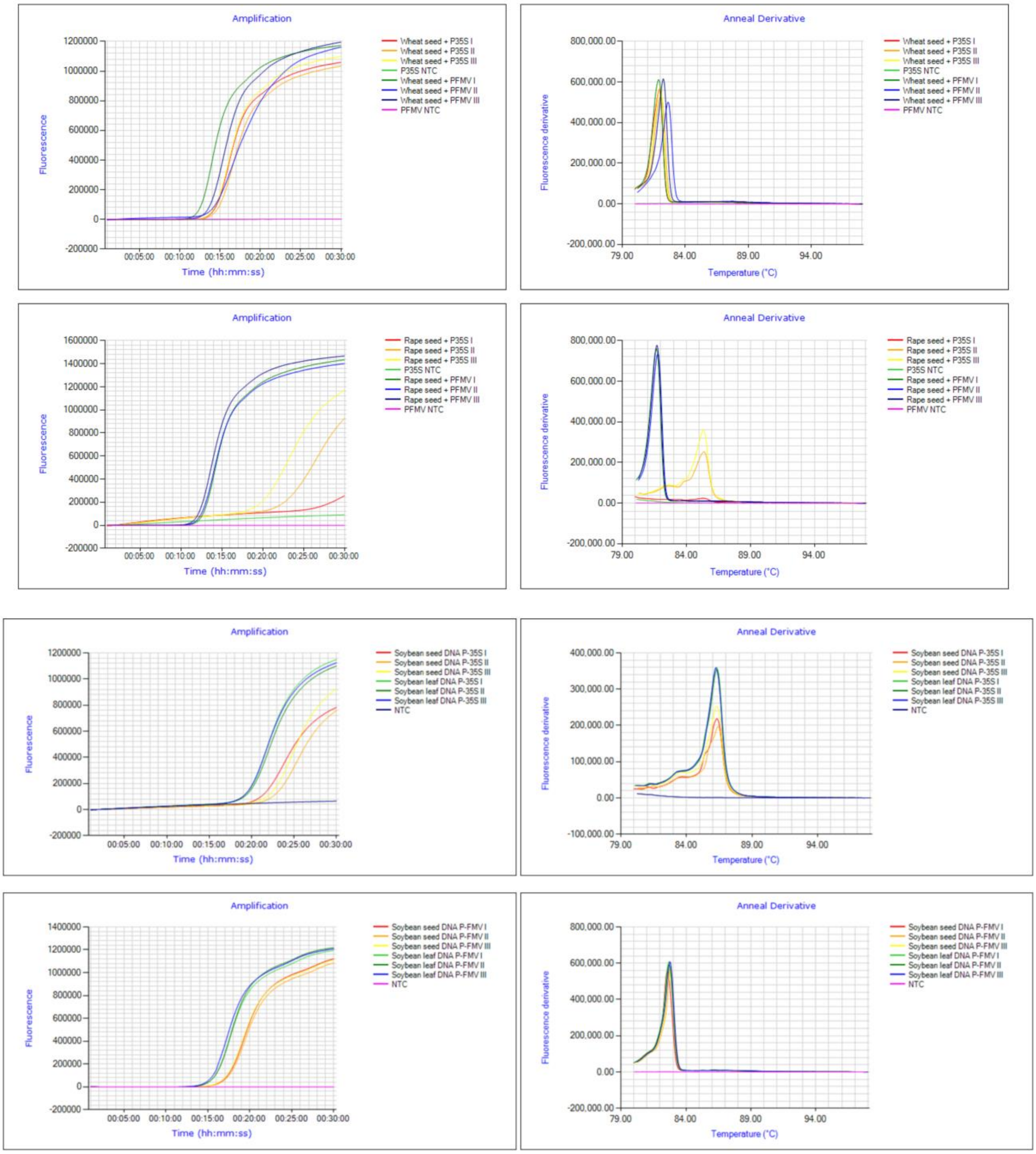

Figure S4. Amplification curves and melting profiles of Real Time LAMP assay for seed and leaf DNA of rape seed, wheat and soybean, extracted with Chelex method and spiked with 0,001 ng/μL gBlock (P-35S/P-FMV). The legend indicates color-coded reactions.

Supplementary Data 2: Nanomaterial Characterization

MXene Characterization

Figure S5a presents the results of $Ti_3C_2T_x$ characterization with XRD and Raman spectroscopy. XRD analysis confirmed the presence of a single-phase $Ti_3C_2T_x$, which indicates the successful etching of the Ti_3AlC_2 MAX phase without its residuals, as could be observed by comparing the two XRD patterns. Sharp and intense peaks indicate a high crystallinity, where the most intense belongs to the 002 crystallographic plane.

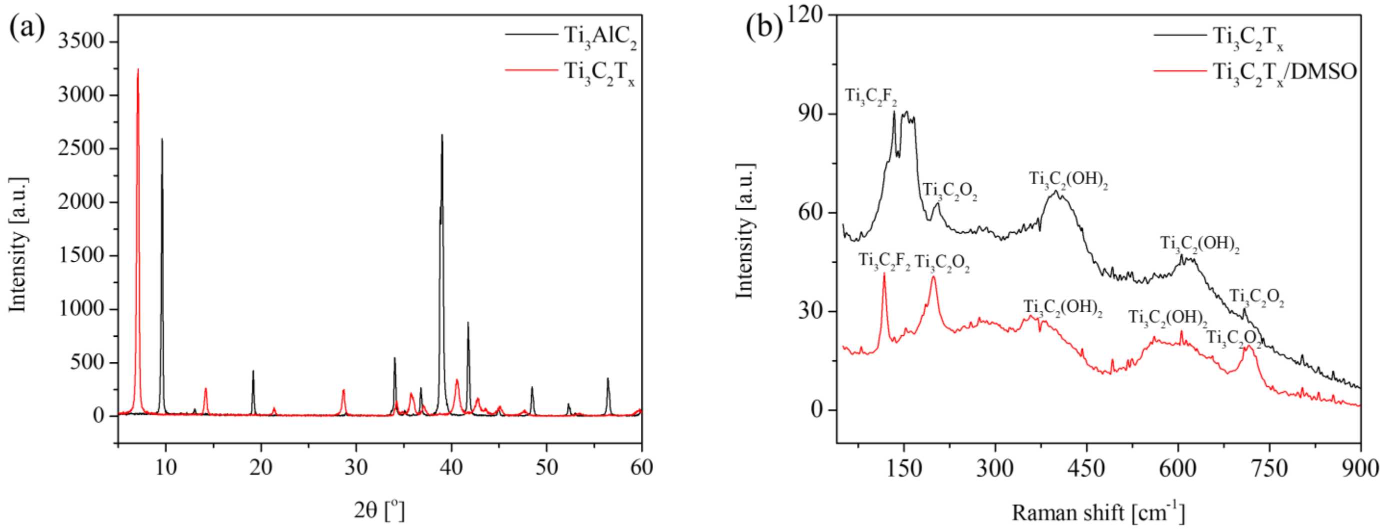

Figure S5. (**a**) XRD spectra of Ti_3AlC_2 MAX phase and parent $Ti_3C_2T_x$ MXene; (**b**) Raman spectra of the obtained MXene compared to the sample exfoliated with DMSO.

Raman spectroscopy is a powerful tool for characterizing the structural and electronic properties of MXenes, providing insights into their phase purity, defect density, and interlayer interactions. In this study, we compare the Raman spectra of an unexfoliated MXene (multilayer) sample with a delaminated (few-layer/single-layer) MXene sample prepared using dimethyl sulfoxide (DMSO) as an intercalant and dispersant (Figure S5b). MXenes, particularly $Ti_3C_2T_x$, exhibit several characteristic Raman-active modes, such as low-frequency modes (< 300 cm^{-1}), related to Ti-Ti and Ti-C vibrations, influenced by interlayer coupling [95,96]. Mid-range modes (200–700 cm^{-1}) are associated with out-of-plane (A_g) and in-plane (E_g) vibrations of Ti and C atoms [97]. The unexfoliated sample shows several peaks below 300 cm^{-1}, corresponding to Ti-Ti vibrations and interlayer interactions. These modes are prominent due to the ordered stacking of multiple layers. The spectrum exhibits a dominant A_{1g} mode near 200–250 cm^{-1}, attributed to out-of-plane vibrations of Ti atoms, which is sensitive to interlayer spacing, together with broadened E_g modes (~400–600 cm^{-1}), arising from in-plane vibrations, indicating some disorder but still retaining long-range order [96,98]. Delamination with DMSO leads to significant spectral changes, such as the suppression of low-frequency modes (< 300 cm^{-1}), particularly the Ti-Ti vibrations, due to weakened interlayer coupling after exfoliation. The shift and broadening of the A_{1g} mode indicate reduced interlayer restoring forces and increased surface effects. Enhanced E_g mode intensity (~400–600 cm^{-1}) suggests greater in-plane vibrational freedom in isolated layers. These findings align with previous studies showing that the mechanical or chemical delamination of MXenes leads to higher surface area and accessibility of active sites (beneficial for energy storage), as well as increased defect density, which can enhance catalytic activity but may reduce electrical conductivity.

The Brunauer–Emmett–Teller (BET) surface area analysis revealed significant differences in the porosity and accessible surface area between pristine $Ti_3C_2T_x$ MXene and its DMSO-delaminated counterpart ($Ti_3C_2T_x$/DMSO). Figure S6 shows the N_2 adsorption–desorption isotherms of $Ti_3C_2T_x$ and $Ti_3C_2T_x$/DMSO samples. The measured BET surface areas were 8.89 m^2/g for the untreated $Ti_3C_2T_x$ and 230.71 m^2/g for the DMSO-delaminated sample, indicating a dramatic increase in surface area after exfoliation. The low surface area (8.89 m^2/g) of the pristine $Ti_3C_2T_x$ suggests that the material exists in a tightly stacked multilayer form, consistent with its unexfoliated state, as seen in SEM images. In contrast, the DMSO-delaminated $Ti_3C_2T_x$ (230.71 m^2/g) exhibits a significant increase in surface area, confirming successful exfoliation into few-layer or monolayer flakes. This expansion

is attributed to the disruption of interlayer interactions and the introduction of open structures with higher porosity. The drastic increase in surface area after DMSO treatment has critical implications for biosensor performance due to enhanced analyte adsorption and improved electrochemical activity. The measured BET surface area of $Ti_3C_2T_x$/DMSO (230.71 m^2/g) aligns well with previously reported values for delaminated $Ti_3C_2T_x$ (~200–300 m^2/g), confirming effective exfoliation [99]. The pristine MXene's low surface area is also consistent with the literature, where non-exfoliated MXenes typically exhibit surface areas below 20 m^2/g [100].

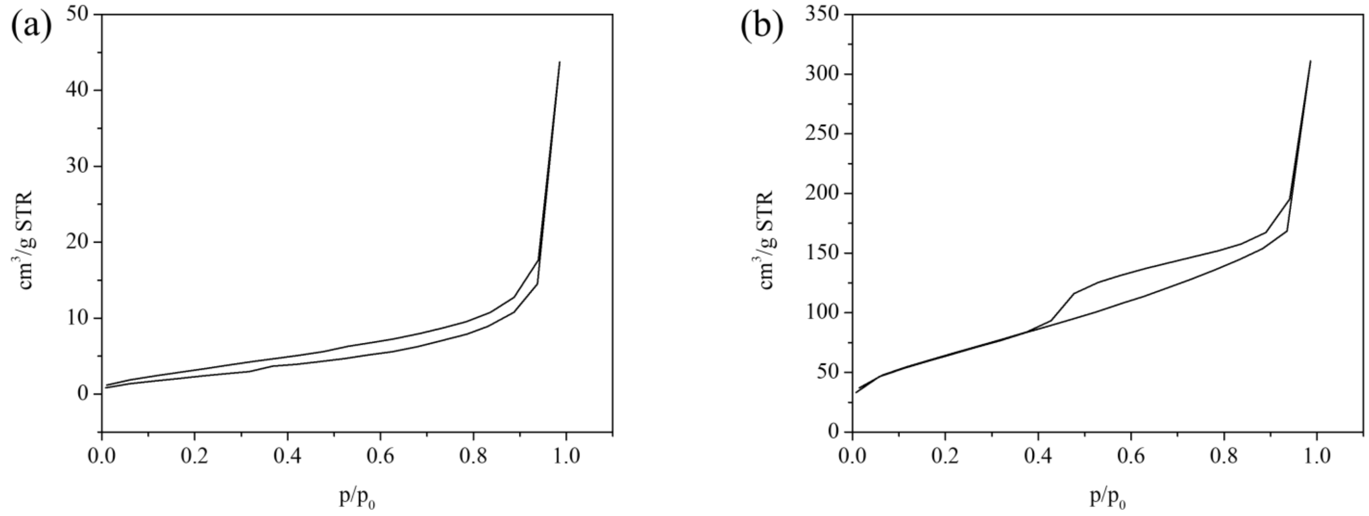

Figure S6. (**a**) N_2 adsorption–desorption isotherms of $Ti_3C_2T_x$ and (**b**) $Ti_3C_2T_x$/DMSO sample.

Supplementary Data 3: Electrochemical Detection

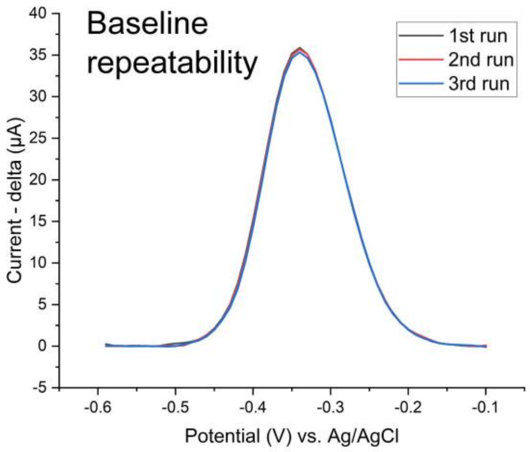

Figure S7. Repeatability of the baseline (1 × PBS) at the same Zensor AUTE100 commercial electrode immobilised with P-35S oligonucleotide linear DNA probes (surface density of the probes 750 pmol/cm^2 with MB); SWV (pulse amplitude 75 mV, pulse width 2 ms, step potential 10 mV, potential window −0.60 to −0.10 V vs. saturated Ag/AgCl).

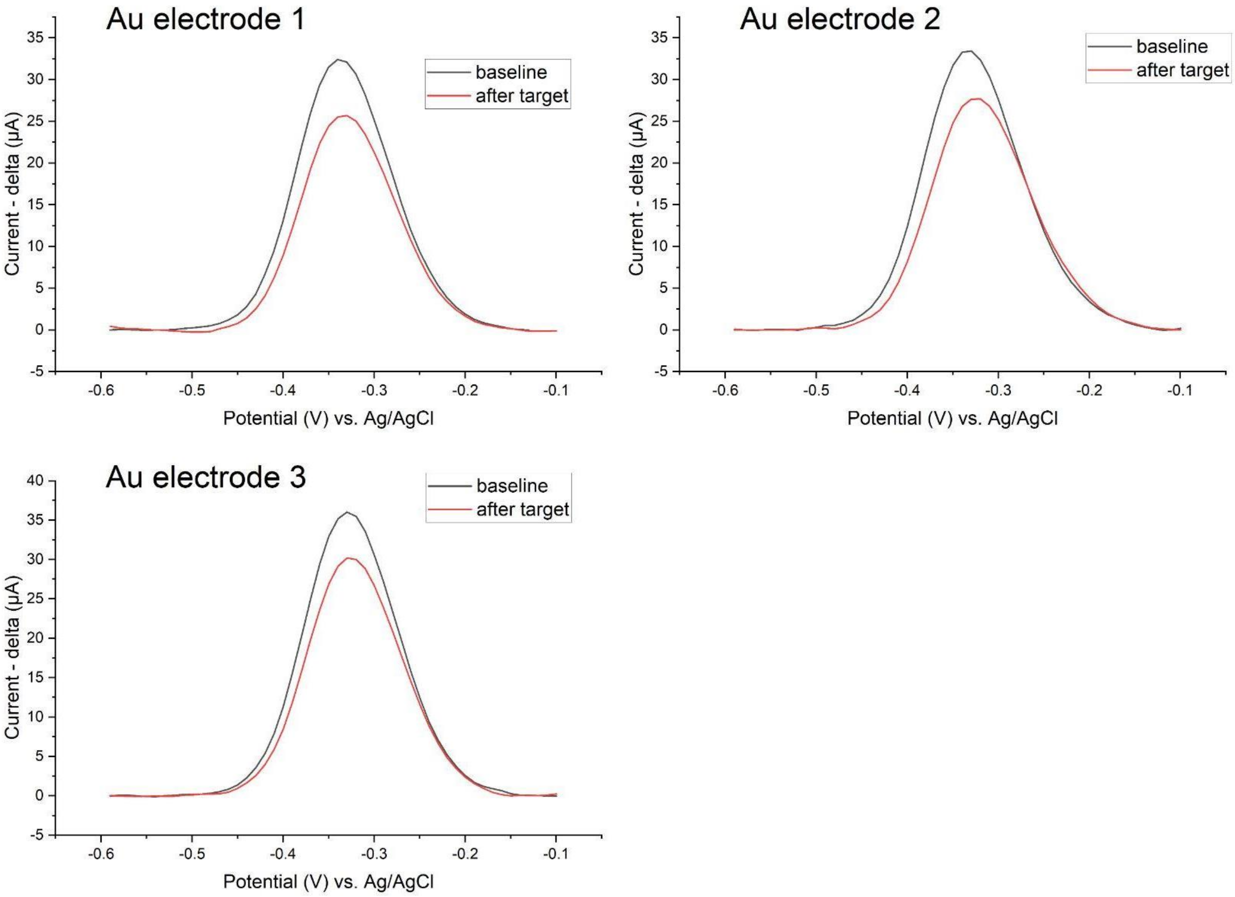

Figure S8. Repeatability of the detection at three different electrodes (commercial screen-printed gold (Zensor AUTE100)). Linear oligonucleotide DNA probes (surface density of 750 pmol/cm^2, P-35S region) carrying methylene blue (MB) were employed and baseline responses (black traces) were recorded in 1× PBS by square-wave voltammetry (pulse amplitude 75 mV, pulse width 2 ms, step potential 10 mV, potential window −0.60 to −0.10 V vs. saturated Ag/AgCl). Red traces depict the signals after 1 h hybridisation with 30 min LAMP reaction products (template gBlock concentration 10^{-3} ng/µL diluted 1:100 in 1 × PBS), measured under the same conditions; detection peak decrease is 5.7 µA, 6.3 µA and 5.8 µA for Au electrodes 1, 2 and 3, respectively, with st. deviation = 0.32.

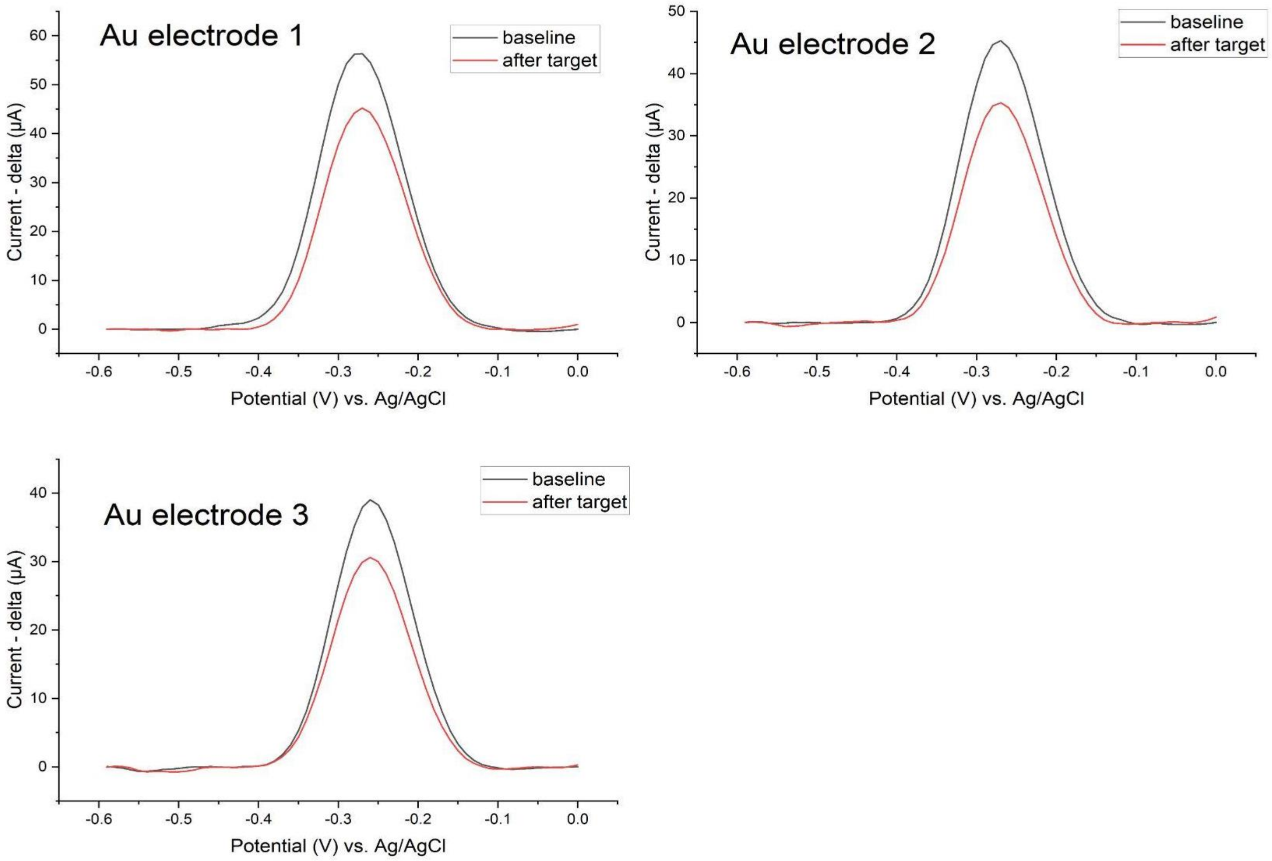

Figure S9. Repeatability of detection at three different electrodes (PVD-coated gold electrodes made in-house); linear oligonucleotide DNA probes (surface density of 750 pmol/cm^2, P-35S region) carrying methylene blue (MB) were employed; baseline responses (black traces) were recorded in 1 x PBS by square-wave voltammetry (pulse amplitude 75 mV, pulse width 2 ms, step potential 10 mV, potential window −0.60 to −0.10 V vs. saturated Ag/AgCl). Red traces depict the signals after 1 h hybridisation with 30 min LAMP reaction products (template gBlock concentration 10^{-3} ng/μL diluted 1:100 in 1 × PBS), measured under the same conditions; detection peak decrease is 11.1 μA, 10.0 μA and 9.6 μA for Au electrodes 1, 2 and 3, respectively, with stDEV of 0.77.

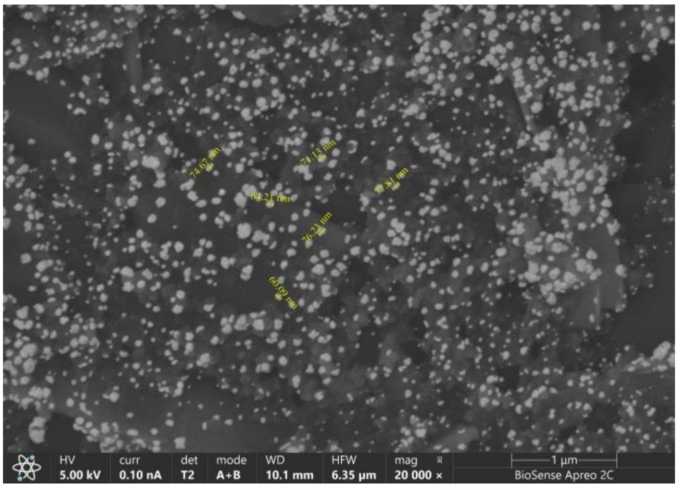

Figure S10. SEM image of Au nanoparticles that were used for signal enhancement.

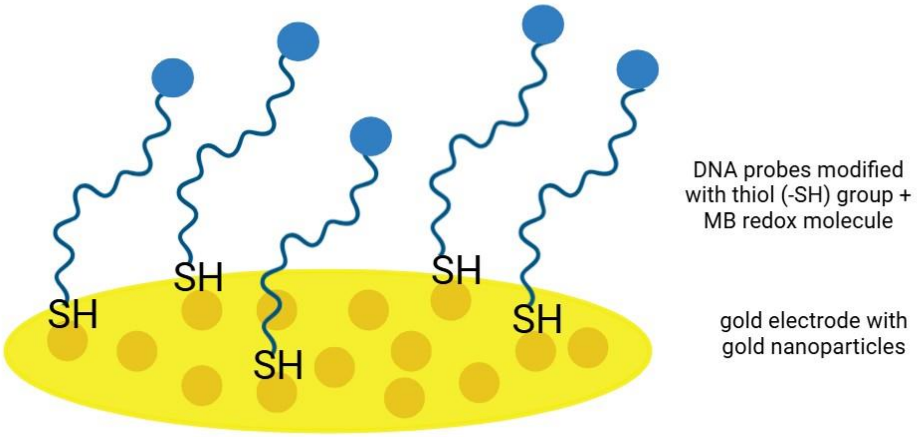

Figure S11. Schematic illustration of a gold electrode modified with gold nanoparticles bearing DNA probes covalently attached via thiol–gold bonds.

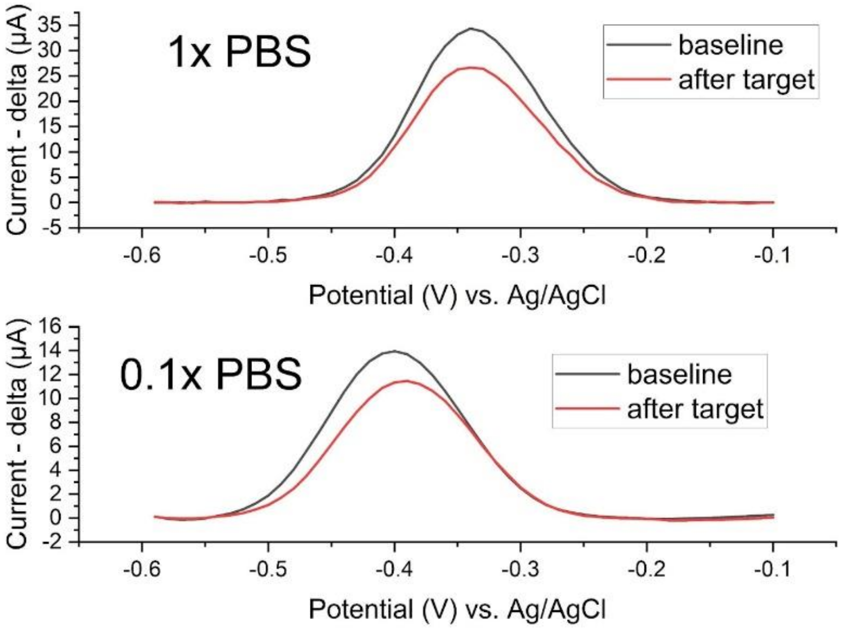

Figure S12. P-35S oligonucleotide DNA probes (surface density 750 pmol/cm^2 with MB) immobilised on Zensor AUTE100 commercial electrodes. A comparison of DNA target detection in 0.1 × PBS and 1 × PBS after 1 h of DNA hybridization detection; square wave voltammetry (pulse amplitude 75 mV, pulse width 2 ms, step potential 10 mV, potential window −0.60 to −0.10 V vs. saturated Ag/AgCl); the detection of target DNA molecules (30 min LAMP products made with 10^{-3} ng/μL of template gBlock, 100× diluted in 1 × PBS after the LAMP reaction).
